# Resiliency of Mutualistic Supplier-Manufacturer Networks

**DOI:** 10.1038/s41598-019-49932-1

**Published:** 2019-09-19

**Authors:** Mengkai Xu, Srinivasan Radhakrishnan, Sagar Kamarthi, Xiaoning Jin

**Affiliations:** Mechanical and Industrial Engineering, Northeastern University, Boston, MA USA

**Keywords:** Scientific data, Complex networks

## Abstract

Current Supplier-Manufacturer (SM) networks are highly complex and susceptible to local and global disruptions, due to connectivity and interdependency among suppliers and manufacturers. Resiliency of supply chains is critical for organizations to remain operational in the face of disruptive events. Existing quantitative analyses oversimplify the mutualistic nature of SM networks, in which failure of individual entities affects not only the directly connected entities but also those connected indirectly. In this work we investigate resiliency of SM networks using the quantitative methods employed to study mutualistic ecological systems. Much like in ecological systems, catastrophic failures of SM networks are difficult to predict due to high dimensionality of their interactive space. To address this, first we create a bipartite representation and generate a multidimensional nonlinear model that captures the dynamics of a SM network. We transform the multidimensional model into a two-dimensional model without sacrificing the model’s ability to predict the point of collapse. We extensively validate the model using real-world global automotive SM networks. We observe that the resiliency of a SM network depends on both the network structure and parameters. The current work offers a means for designing resilient supply chains that can remain robust to local and global perturbations.

## Introduction

Large manufacturing organizations rely on many suppliers spread across globally to acquire parts for their products. At factory level, internal perturbations in the form of labor unrest, machine reliability issues, or material shortage affect the optimum performance of a factory and the associated SM network. At meso level, geographical conditions surrounding the factory, local perturbations in the form of natural disasters, material shortage or restrictive economic policies tend to affect the performance of suppliers and manufacturers. Finally, at enterprise level, global perturbations in the form of reduction in overall demand or weak economic markets reverberate across the entire supply chain. Disruptions in supply chain can be detrimental to a firm’s performance and can negatively impact the overall financial gains^1–5^. Organizations are proactively paying attention to improving their Supply Chain Resiliency (SCR)^5–8^. SCR enables SM networks to withstand disruptions at multiple scales to mitigate performance and economic losses^5^. For example, it was SCR that enabled Japanese firms to rapidly respond and restore production post a massive earthquake in 2011 through resilient resource mobilization^5,9^. The common notion of SCR is the ability of a supply chain to cope with the unavoidable and unpredictable performance disruptions and bounce back to the original operating state or to a new desirable operating state^7,10–14^.

The existing studies on resiliency of supply chains focus on both qualitative and quantitative approaches, though qualitative ones are more common. These strategies involve heuristics and rule of thumb strategies built on past experience. At factory level, redundancy in the form of parallel machines and storage buffers improves resiliency^15^. At system level, the risks of supplier disruptions are mitigated by building redundancy through multisource strategies. The common approach in all the qualitative studies is identifying vulnerabilities and building capabilities to counter the vulnerabilities^16^. Quantitative approaches on the other hand focus on operational resiliency. They monitor post-disruption performance of supply chains using key performance metrics (KPIs)^17,18^. The reactive and preventive strategies for improving resiliency minimize the related costs including the cost associated with recovery and performance loss^17^. SM networks are multidimensional, and the existing low-dimensional analyses of SCR do not effectively incorporate structural information of interconnected components and do not sufficiently consider the dependencies between the suppliers and manufacturers. Several studies have investigated the robustness of highly interconnected complex networks in the event of disruption^19–22^. Novel strategies are proposed to deal with cascading failures in engineered connected systems such as power grid, and communication networks^21,22^. Supply chains have been investigated as a complex adaptive system^23^. This allows researchers to model supply chains as a network and analyze them using techniques from the field of nonlinear dynamics, statistical physics and information theory. However, current supply chain network models do not consider the mutualistic interaction effects of supplier and manufacturer on each other. The phenomenon of mutualism creates an environment in which two different groups benefit mutually due to interactions among members across the groups. The existing analysis only focuses on manufacturer’s resiliency as a function of supplier disruptions. However, because of mutualism, the survival (and growth) of one manufacturer positively influences other manufacturers, and similarly the survival (and growth) of one supplier positively influences other suppliers. Intuitively speaking a much better configuration to study SCR would be to treat manufacturers and suppliers as different classes having between-class interactions. Such configurations are commonly investigated in mutualistic ecological networks. The dynamical properties of such mutualistic systems are represented by a mathematical model that combines the network structure and the parameters to assess the resiliency of the system against perturbations^24–26^. The mathematical models presented in these studies involve growth, decay, competition, and mutualistic effects. Previous studies have reduced the multidimensional models into one- or two-dimensional models to effectively represent the resiliency function^24,25^. In such dimension–reduced model the resiliency of the system depends on the network structure and parameters alone. We believe that the same analysis can be extended to SM networks where one can treat the manufacturers and suppliers as different species who benefit from mutualistic interactions. The dynamics that govern the abundance of a species in a mutualistic ecological network share similarity with the abundance of throughput (production quantity) in a SM network. Similar to ecological systems, SM networks include a growth component which is bounded by the maximum capacity of the production center, decays in form of internal reliability loss that reduces the production quantity, competition in the form of pricing and technology, and finally the mutualistic effects can be captured using the network structure. Apart from the similarity in the dynamics, interestingly, the structural properties of ecological networks and SM networks are strikingly similar^27–29^. These provide an impetus to emulate the approaches used for ecological networks.

First, we formulate a dynamical model for the multidimensional SM network combining structural information and network parameters. Next, we reduce the multidimensional formulation to an effective two-dimensional model. Using this dimension-reduced model, we introduce local and global perturbations and assess their impact on the throughput of the SM network. We observe that the dimension-reduced model, like multidimensional model, is insensitive to external noise and parameter variations, and is able to generate the same point of collapse as the one generated by the multidimensional model. In addition, we observe that the SM network resiliency is highly sensitive to the network structural properties, namely, nestedness and density. The proposed approach is accurate in identifying the point of collapse (tipping point) beyond which a SM network will be unable to deliver the desired throughput and unable to regain its production capacity.

## Results

### Nonlinear dynamical model of SM mutualistic network

**Figure 1:**
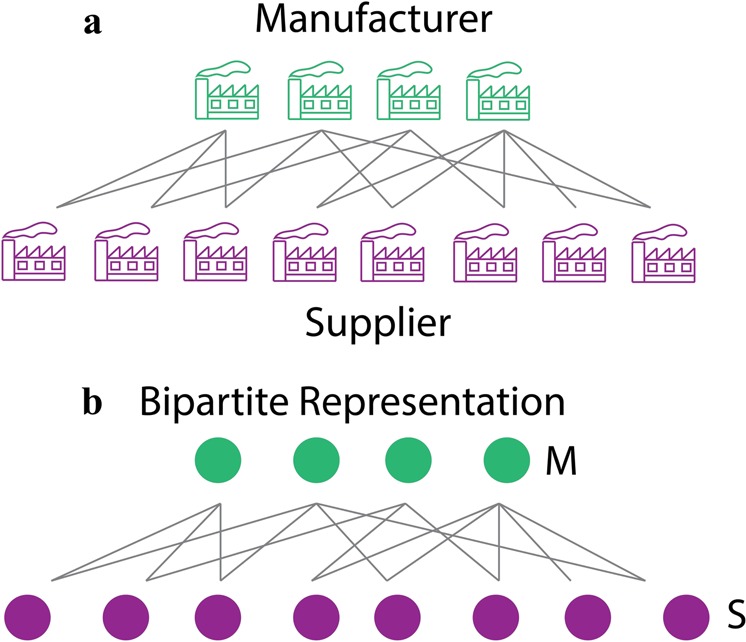
(**a**) A mutualistic Supplier-Manufacturer (SM) network, (**b**) bipartite representation of SM network.

Figure 1a shows a mutulistic SM network, and Figure 1b shows the bipartite representation of the SM network. The manufacturers and the suppliers are represented as nodes. In Figure 1b, *M* represents a manufacturer and *S* represents a supplier. A manufacturer is connected to a supplier if the supplier provides parts/products to the manufacturer. The link is unweighted, i.e., even if the supplier supplies multiple components to the manufacturer, the link will still have a weight of one.

Mathematically the bipartite network is represented as adjacency matrix *A* whose element *y*_*ij*_ is 1 if manufacturer *i* is connected to supplier *j*; 0 otherwise. Equations (1) and (2) represent the dynamics of a mutualistic SM network. While Eq. (1) models the nonlinear dynamic change of the throughput of manufacturer *i*, Eq. (2) similarly models the nonlinear dynamic change of the throughput of supplier *i*.

1 $$\begin{array}{rcl}\frac{d{M}_{i}}{dt} & = & \mathop{\underbrace{{M}_{i}(1-\frac{{M}_{i}}{{{K}_{i}}^{(M)}})}}\limits_{{\rm{Growth}}}-\mathop{\underbrace{{\alpha }_{i}^{(M)}{M}_{i}}}\limits_{{\rm{Internal}}\,{\rm{perturbation}}}\\  &  & -\,\mathop{\underbrace{{M}_{i}\,\mathop{\sum }\limits_{j=1,j\ne i}^{{Q}_{M}}\,{\beta }_{ij}^{(M1)}{M}_{j}-{M}_{i}\,\mathop{\sum }\limits_{j=1,j\ne i}^{{Q}_{M}}\,{\beta }_{ij}^{(M2)}{M}_{j}}}\limits_{{\rm{Competition}}}\\  &  & +\,\mathop{\underbrace{{M}_{i}\frac{{\sum }_{k=1}^{{Q}_{S}}\,{\gamma }_{ik}^{(M)}{S}_{k}}{1+h\,{\sum }_{k=1}^{{Q}_{S}}\,{\gamma }_{ik}^{(M)}{S}_{k}}}}\limits_{{\rm{Mutualistic}}\,{\rm{interaction}}}+{\mu }_{i}^{(M)}\end{array}$$

2 $$\begin{array}{rcl}\frac{d{S}_{i}}{dt} & = & {S}_{i}(1-\frac{{S}_{i}}{{K}_{i}^{(S)}})-{\alpha }_{i}^{(S)}{S}_{i}-{S}_{i}\,\mathop{\sum }\limits_{j=1,j\ne i}^{{Q}_{S}}\,{\beta }_{ij}^{(S1)}{S}_{j}\\  &  & -\,{S}_{i}\,\mathop{\sum }\limits_{j=1,j\ne i}^{{Q}_{S}}\,{\beta }_{ij}^{(S2)}{S}_{j}+{S}_{i}\frac{{\sum }_{k=1}^{{Q}_{M}}\,{\gamma }_{ik}^{(S)}{M}_{k}}{1+h\,{\sum }_{k=1}^{{Q}_{M}}\,{\gamma }_{ik}^{(S)}{M}_{k}}+{\mu }_{i}^{(S)}\end{array}$$

Eq. (1) follows the proposed logistic growth model for our SM network application. Here *M*_*i*_ is the throughput of manufacturer *i*; ${K}_{i}^{(M)}$ is the production capacity of manufacturer *i* acting as plant capacity (i.e., capacity limiting term)^30–33^; ${\alpha }_{i}^{(M)}$ is an internal perturbation parameter; ${\beta }_{ij}^{(M\mathrm{1)}}$ and ${\beta }_{ij}^{(M\mathrm{2)}}$ are the effects of price competition and technology competition between manufacturers *i* and *j*; *Q*_*M*_ is the number of manufacturers; ${\gamma }_{ik}^{(M)}$ represents the mutualistic interaction effect between manufacturer *i* and supplier *k*; the saturation parameter *h* is used to characterize the squashing effect; *Q*_*S*_ is the number of suppliers; and ${\mu }_{i}^{(M)}$ indicates the production subcontracting/outsourcing intensity. Same notations are applied to Eq. (2) except that the superscript stands for suppliers.

#### Growth term

**Figure 2:**
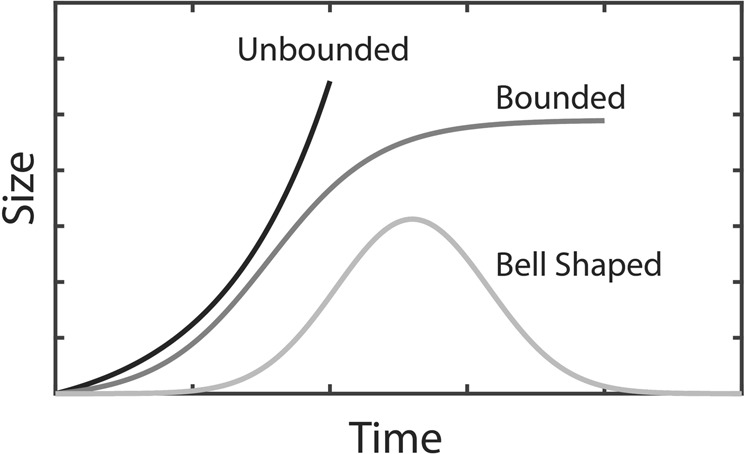
Commonly explored growth models.

The first term on the right-hand side of Eq. (1) represents the growth term. In this research we are measuring growth of a manufacturer or supplier in terms of throughput (production volume). Figure 2 shows the commonly adopted growth models, which are either unbounded, bounded, and bell shaped curves. A bell shaped growth model will fail to represent the realty that when manufacturers decommission specific product lines, they compensate for the production loss at the enterprise level by producing other products. A bell shape growth model for the proposed approach will indicate that, over time the throughput of a manufacturer declines to zero (which translates to extinction of manufacturer) after reaching a peak production.

In this context, an unbounded (e.g., exponential) growth model indicates that throughput keeps increasing for ever without a limit. The unbounded growth models, although mathematically appealing, they are not supported by empirical evidence^34^. For a given plant capacity, the unbounded growth model is feasible only when the throughput of a manufacturing plant scales linearly or super-linearly with the plant utilization, which is not the case in reality. In practice, when the plant utilization increases, the throughput of the plant also increases. However, beyond a certain point, any increase in utilization is followed by a decrease in the throughput. The root cause for this trend is that the increase in utilization comes at the expense of avoiding equipment maintenance which adversely affects machine reliability. This in turn leads to increase in the frequency of failures (*F*) and longer repair time (*T*_*d*_). An increase in *T*_*d*_ and/or *F* increases downtime, work in progress (WIP), and cycle time (*T*_*c*_)^31,32^. The fact that throughput does not scale linearly with the plant utilization is not represented correctly in the unbounded growth model.

Therefore, a bounded growth model, especially a logistic shape, can better represent throughput growth pattern of a manufacturer or a supplier. In a bounded growth model, the growth becomes bounded as a consequence of the growth itself. Constraints such as space limit, finite talent pool, policies, regulations, resources (water and energy), and the risk of unmanageable complexity, make unending growth untenable. Logistic growth models have extensively been investigated for ecological systems where the environment carrying capacity, and competition act as growth limiting factors^35,36^. In addition, logistic growth behavior has been observed in new car registrations in Japan^37^, car population in Italy^37^, and annual production of natural resources^34,38^.

#### Internal perturbation term

The second term in Eq. (1), *α*_*i*_, is an internal perturbation parameter. It represents the production system reliability which is adversely affected by internal perturbations such as machine failure and labor unrest^39–42^. Throughput nonlinearly decreases as the production system reliability decreases^39–42^. When the throughput is high, a small reduction in reliability due to internal perturbation could result in large throughput reduction; when the throughput is small, a small reduction in reliability can only cause a minor change in throughput^39–42^.

#### Price and technology competition terms

The third and the fourth terms in Eq. (1) reflect the effect of price competition and technology competition between manufacturers (suppliers) *i* and *j* on their throughput^43–45^. The throughput of a manufacturer (supplier) declines as the competition becomes fierce. When ${\beta }_{ij}^{(1)} > {\beta }_{ij}^{(2)}$, the price competition dominates the technological advantage. The dominance is reversed when ${\beta }_{ij}^{(2)} > {\beta }_{ij}^{(1)}$.

#### Mutualistic interaction term

The fifth term in Eq. (1), ${\gamma }_{ik}$, captures the mutualistic interaction effect between manufacturer *i* and supplier *k*. When both manufacturers and suppliers are generating high throughput, the interaction effect between them saturates the throughput growth^24,25,46^. The saturation constant *h* is used to characterize the squashing effect. The term ${\gamma }_{ik}$ is defined as^26,47^

3 $${\gamma }_{ik}={y}_{ik}\frac{{\gamma }_{0}}{{({N}_{i})}^{\delta }}$$

where ${y}_{ik}=1$ if manufacturer *i* and supplier *k* interact; ${y}_{ik}=0$ otherwise. The term *γ*_0_ represents the level of mutualistic strength, which in this work is set to 1. The term *δ* denotes the mutualistic trade-off modulating the relation between the interaction strength and the number of interactions *N*_*i*_. The interaction strength *γ*_*ik*_ is determined by the node degree of manufacturer *i*. The more connections manufacturer *i* maintains, the weaker is the mutualistic interaction between manufacturer *i* and supplier *k*. This also indicates that when manufacturer *i* partners with a large number of suppliers, the contribution of each of these suppliers will be smaller.

#### Outsourcing intensity term

In Eq. (1), *μ* indicates the production subcontracting/outsourcing intensity. In the long run, *μ* will have a negligible effect on a manufacturer’s dynamics, but in the short term, *μ* exerts a considerable influence. However in reality, manufacturers and suppliers rarely outsource their proprietary components/products for the fear of creating unintended opportunities for future competition. So even for short term, one can assume a negligible effect of *μ* on throughput of a SM network.

As mentioned earlier, the nonlinear dynamics of suppliers are modeled by Eq. (2). All the terms in Eq. 2 have the same interpretation for suppliers as they have for manufacturers in Eq. (1).

**Dimension reduction**

4 $$\begin{array}{rcl}\frac{d{M}_{eff}}{dt} & = & {M}_{eff}(1-\frac{{M}_{eff}}{K})-\alpha {M}_{eff}-{\beta }^{(M)}{({M}_{eff})}^{2}\\  &  & +\,{M}_{eff}\frac{\langle {\gamma }_{M}\rangle {S}_{eff}}{1+h\langle {\gamma }_{M}\rangle {S}_{eff}}+\mu \end{array}$$

5 $$\frac{d{S}_{eff}}{dt}={S}_{eff}(1-\frac{{S}_{eff}}{K})-\alpha {S}_{eff}-{\beta }^{(S)}{({S}_{eff})}^{2}+{S}_{eff}\frac{\langle {\gamma }_{S}\rangle {M}_{eff}}{1+h\langle {\gamma }_{S}\rangle {M}_{eff}}+\mu $$

Equations (1) and (2) represent a multidimensional SM network, where each manufacturer or each supplier is associated with its own dynamics. Multidimensionality increases the computational load and is lack of predictability, and those issues can be resolved by reducing the dimensionality of the model described by Eqs 1 and 2. The dimension-reduced models are given in Eqs (4) and (5). They can be used to capture the SM network’s dynamics and topological information that was described by Eqs (1) and (2). The dimension reduction procedures are given in the Supplementary Information Sections 1–3. In Eqs (4) and (5), *M*_*eff*_ and *S*_*eff*_ represent the effective average throughput of the SM network aggregated over all manufacturers and suppliers respectively; *K* is the production capacity; *α* is the effective internal perturbation parameter; *β* is the effective competition parameter that combines both the price and technology competitions; and $\langle {\gamma }_{M}\rangle$ and $\langle {\gamma }_{S}\rangle$ are the effective mutulistic strengths for manufacturers and suppliers. The terms $\langle {\gamma }_{M}\rangle$ and $\langle {\gamma }_{S}\rangle$ can be computed using averaging methods such as unweighted average, degree-weighted average, and eigenvector-based average^25^. In this work we consider the degree-weighted average method for computing $\langle {\gamma }_{M}\rangle$ and $\langle {\gamma }_{S}\rangle$, since it is robust against noise and parameter variations^25^.

In the following sections we investigate the effect of network structure on the resiliency function, demonstrate the robustness of the dimension-reduced model against ransom noise and random parameter variations, and discuss the ability of the dimension-reduced model to predict the point of collapse in the structure-parameter space.

### Analysis of real world SM networks

Studies have revealed a strong similarity between structural properties of mutualistic ecological networks and SM networks^27–29^. Network properties, mainly, nestedness, degree distribution, and modularity are significantly similar for ecological networks and SM networks^27^. Nestedness is a property of a bipartite network where specialists (e.g., pollinators that visit a few specific type of plants) interact with species of other class with whom generalists (e.g., pollinators that visit many types of plants) interact^48^. Similarly SM networks are characterized by the presence of “generalist” and “specialist” suppliers^29^. The generalists have the technical capabilities to manufacture a wide variety of products at lower volumes (job shop production) while specialists have the capabilities to manufacture products with low design variation at higher volumes (mass production). The nestedness in SM networks aligns with that of ecological networks where, the specialist suppliers interact with manufacturers with whom the generalist suppliers interact^29^. This redundancy (nestedness) ensures the resiliency of SM networks, which otherwise will fail with the demise of a few suppliers. Apart from the nested property, network density plays a major role in ensuring the resiliency of a mutualistic system^24^.

We validate the models proposed in Eqs (4) and (5) using the global automotive supply chain network data. There are 376 manufacturers and 5229 suppliers who supply approximately 300 components including critical and noncritical components. The critical components like piston and connecting rod are essential for the function of an automobile. The noncritical components like lamp wiper and exterior are required although their failures do not affect the primary function of an automobile. The data spans from 1999 to 2020 (future orders are also considered). We divide the automotive SM networks into 21 sub-networks according to AA classification scheme as given in Supplementary Table S1. Sub-networks are considered for two reasons: (a) automotive assemblies are highly modular and independent of each other (e.g., an engine sub-assembly has no dependence on a door panel for its production completion), and (b) to allow simulation runs to complete in a reasonable amount of time. We create two versions of automotive SM networks for analysis. In the first one, we consider all the manufacturers and suppliers across multiple years (longitudinal data). In such networks, manufacturers do not always get parts/products from the same suppliers every year. We form 21 longitudinal networks corresponding to each of the 21 subnetworks. The network properties (densisty and nestedness) for longitudinal networks are given in Supplementary Table S2. In the second version of networks, we consider two automotive SM subnetworks just for the year 2017 (cross sectional data). The network properties (densisty and nestedness) for cross sectional networks are given in Supplementary Table S3. Resiliency patterns of networks using cross sectional data show the point-in-time (or current) resiliency of the automotive SM networks. The networks formed using longitudinal data are not useful to assess the true resiliency of the networks compared to the networks formed using the cross sectional data. However, the networks formed using longitudinal data serve as an ideal reference for evaluating resiliency of the network that can be obtained using networks built with the cross sectional data.

In tune with the previous studies^27–29^, we observe that automotive SM networks generated using both longitudinal and cross sectional data exhibit high nestedness and low network density (see Supplementary Information Tables S2 and S3), striking similarity to the network properties exhibited by ecological networks. It is not unusual for automotive SM networks to exhibit such network properties. It is in the best interest of manufacturers to have many suppliers, while this is constrained by the supplier capabilities and production economics. A supplier may take many years to upgrade technology and skills to align with the manufacturer’s requirements. Even though that there are many capable suppliers, manufacturers will work only with optimum number of suppliers to allow suppliers to derive the benefit of economies of scale. In the event of low capacity utilization at the supplier end, the costs cannot be maintained competitive which hampers the survivability of the suppliers. Therefore a network with the structure of low density and high nestedness is created.

#### Effect of network structure

In order to reduce the time complexity of rewiring real world SM networks to understand the effects of nestedness, we use simulated data (having same nestedness and density properties of real world networks) to observe the structural effects on the resiliency of the SM network. We simulate synthetic SM networks with 10 manufacturers and 26 suppliers. The network density, $\bar{a}$, of a bipartite network is computed as $\bar{a}=\frac{m}{I\times J}$, where *m* is the total number of interactions for all the nodes in the SM network, *I* is the number of manufacturers and *J* is the number of suppliers^49^. The value of density varies from 0 to 1, 0 being low density and 1 being high density. The nestedness is computed using the NTC method^50^. The normalized value of nestedness varies form 0 to 1, 0 being low nestedness and 1 being high nestedness.

We remove *f*_*n*_ fraction of suppliers and observe the effects of the perturbation on the throughput of suppliers. Similarly, we remove *f*_*n*_ fraction of manufacturers and observe the effects of the perturbation on the throughput of manufacturers. We remove *f*_*l*_ fraction of suppliers and see the effect of perturbation on manufacturer throughput, and finally we remove *f*_*l*_ fraction of manufacturers and see the effect of perturbation on supplier throughput. In addition to local disturbances, we also introduce global perturbations by reducing *f*_*w*_ which is the fraction of interaction strength loss in Eqs (4) and (5). For all the perturbations, the effective throughput of the manufacturers and suppliers are computed using Eqs (4) and (5). We assume that in the event of a node or link loss the manufacturers do not redistribute their workload among the remaining suppliers and suppliers do not form a new link with remaining manufacturers. It is a realistic assumption considering that the last minute load distribution by manufacturers is impeded by the capacity constraints of the suppliers and, demand and product constraints prevent suppliers to form links with new manufacturers in a very short period of time.

Supplementary Figure S3 shows the result of the SM network resiliency under varying degree of nestedness with constant density. Two networks consisting of 10 manufacturers and 26 suppliers are generated with low (0.19) and high (0.95) nestedness properties. The density is maintained at a constant value of 0.30. Supplementary Figure S4 shows the result of the SM network resiliency under varying degree of density and constant nestedness. The simulation process remains the same as described above except that the two networks are generated with high density (0.68), and low density (0.30). The nestedness value is kept constant at 0.95. First, we observe that irrespective of the network structure, the system is more sensitive to global perturbations than the local perturbations. The network resiliency is sensitive to both nestedness and the network density. High values of nestedness and network density bestow a supply chain with better resiliency. This result is in agreement with the observations for mutualistic ecological networks, where the nested structure allows a resilient system that can accommodate node or link deletion with minimal performance loss^51^. To better understand the relation between network density and nestdeness, a sensitivity analysis is conducted on a synthetic network (10 manufacturers, 26 suppliers) by varying degree of nestedness and network density. The results (see Supplementary Figures S5 to S7) confirm that both nestedness and density contribute to the network resiliency. When the network density is relatively high, the network resiliency is less sensitive to degree of nestedness. Similarly when the degree of nestedness is high, the network resiliency becomes robust to degree of density. Hence either high degree of nestedness or high network density can guarantee the resiliency of the system.

#### Robustness against noise and parameter variation

**Figure 3:**
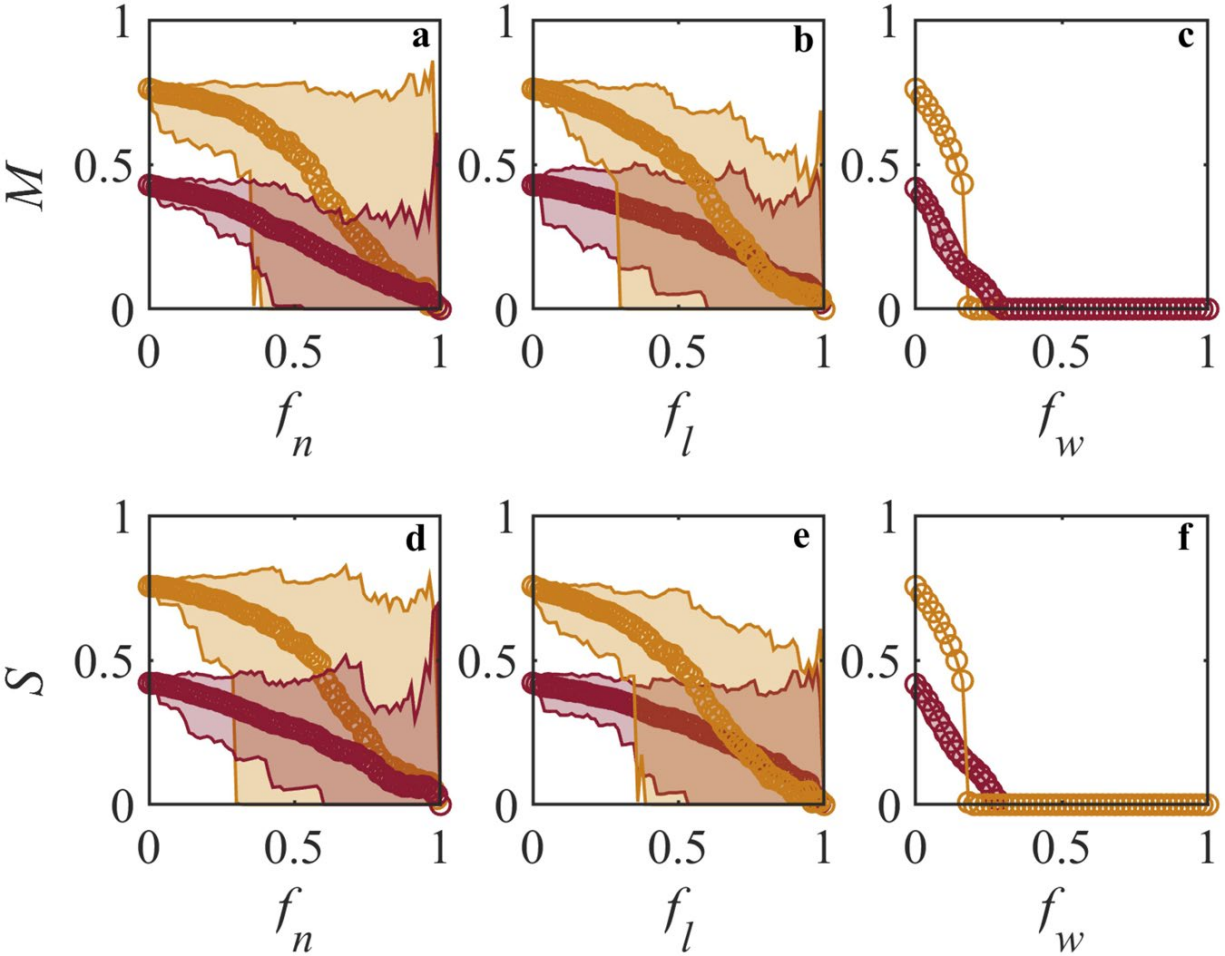
Dynamics of manufacturer and supplier throughputs when subjected to local and global perturbations. The network considered here is for suppliers who supplied drive train components to the manufacturers for the year 2017. It has 75 manufacturers and 80 suppliers. The nestedness value is 0.9703 and the density value is 0.0323. (**a**,**d**) Show the throughputs for manufacturer and supplier when subjected to manufacturer removal *f*_*n*_ and supplier removal *f*_*n*_ respectively. (**b**,**e**) Show the throughputs for manufacturer and supplier when subjected to supplier removal *f*_*l*_ and manufacturer removal *f*_*l*_ respectively. (**c**,**f**) Represent the effect of global perturbation on throughputs of manufacturer and supplier respectively in form of weight reduction *f*_*w*_. Maroon color curves are the results of multidimensional model (Eqs (1) and (2)), Orange color curves are the results of dimension-reduced model (Eqs (4) and (5)). The line with circle is the average of 100 realizations and the shaded area shows the lower bound and the upper bound values. $K=1$, $\alpha =1.2$, $\beta =0.0001$, ${\gamma }_{0}=1$, $\delta =0.5$.

We perform numerical analyses using the multidimensional model (Eqs (1) and (2)) and dimension-reduced model (Eqs (4) and (5)) to examine the sensitivity of the dimension-reduced model to noise and parameter variations, i.e., we examine if the dimension-reduced model is able to predict the point of collapse (point where the SM network collapses unrecoverably) without large deviation. We use a real world SM network with 80 suppliers who supplied drive train parts to 75 manufacturers for the year 2017. The nestedness of the network is 0.9703 and the density is 0.0323. Figure 3 shows the throughput of manufacturers and the suppliers when subjected to local and global perturbations. The parameters for both the multidimensional model and the dimension-reduced model remain the same. The perturbations are introduced randomly and one hundred realizations of each perturbation scenario are performed. We observe that the dimension-reduced model effectively captures the resiliency pattern of the multidimensional model without a significant deviation. The average throughput of both the models converge almost at the same point of collapse.

We then add Gaussian noise of strength 0.1 to the multidimensional model and the dimension-reduced model. We observe that both the models are able to converge at the point of collapse (see Supplementary Figure S8). The observation also extends to scenarios with parameter variation, where a random variation (*U*$[0.8,1.2]$) in value of *K* does not significantly alter the point of collapse for both models (see Supplementary Figure S9). We also introduce random variations in competition parameter *β* using uniform distribution *U*$[0.001,0.002]$ and internal reliability *α* using uniform distribution *U*$[1,1.2]$. We observe that the variations in *α* and *β* do not affect the ability of the dimension-reduced model to predict the point of collapse (see Supplementary Figures S10 and S11). Overall, the effective dimension-reduced model does not deviate from the multidimensional model for predicting the point of collapse and can be used to represent the dynamics of the SM network. For generalizability, we also test the effectiveness of dimension-reduced model using synthetic network. We generate a network of 10 manufacturers and 26 suppliers with a nestedness of 0.95 and a density of 0.19. The observations do not deviate from the earlier investigation done on real world networks and the effective dimension-reduced model does not deviate from the multidimensional model for predicting the point of collapse and can be used to represent the dynamics of the SM network. The results are shown in Supplementary Figures S12 to S15.

### Predicting point of collapse in real world SM networks

**Figure 4:**
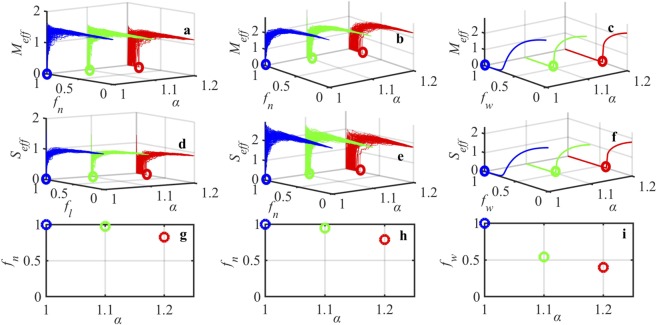
Emergence of point of collapse for network 12 (Supplementary Information Table S2) that considers the main components related to Engine from 2006–2019 (longitudinal data). (**a**) Resilience function *M*_*eff*_ vs. manufacturer removal *f*_*n*_ with the parameter regime of *α*, (**b**) resilience function *M*_*eff*_ vs. manufacturer link loss *f*_*l*_ with the parameter regime of *α*, (**c**) resilience function *M*_*eff*_ vs. global weight loss *f*_*w*_ with the parameter regime of *α*, (**d**) resilience function *S*_*eff*_ vs. supplier link loss *f*_*l*_ with the parameter regime of *α*, (**e**) resilience function *S*_*eff*_ vs. supplier removal *f*_*n*_ with the parameter regime of *α*, (**f**) resilience function *S*_*eff*_ vs. global weight loss *f*_*w*_ with the parameter regime of *α*, (**g**) emergence of the point of collapse computed in terms of manufacturer removal *f*_*n*_ and variation of *α*, (**h**) emergence of the point of collapse computed in terms of supplier removal *f*_*n*_ and variation of *α*, and (**i**) emergence of the point of collapse computed in terms of global weight loss *f*_*w*_ and variation of *α*.

**Figure 5:**
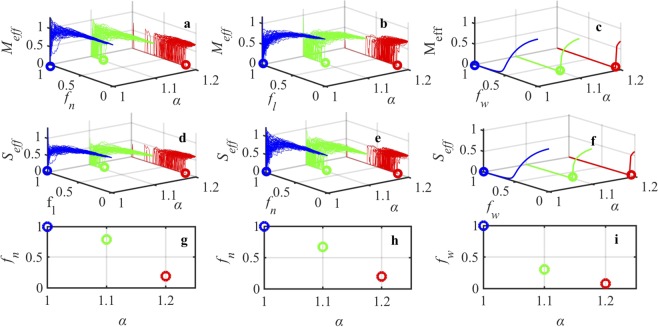
Emergence of point of collapse for network 12 (Supplementary Information Table S3) that considers the main components related to Engine from 2017 (cross sectional data). (**a**) Resilience function *M*_*eff*_ vs. manufacturer removal *f*_*n*_ with the parameter regime of *α*, (**b**) resilience function *M*_*eff*_ vs. manufacturer link loss *f*_*l*_ with the parameter regime of *α*, (**c**) resilience function *M*_*eff*_ vs. global weight loss *f*_*w*_ with the parameter regime of *α*, (**d**) resilience function *S*_*eff*_ vs. supplier link loss *f*_*l*_ with the parameter regime of *α*, (**e**) resilience function *S*_*eff*_ vs. supplier removal *f*_*n*_ with the parameter regime of *α*, (**f**) resilience function *S*_*eff*_ vs. global weight loss *f*_*w*_ with the parameter regime of *α*, (**g**) emergence of the point of collapse computed in terms of manufacturer removal *f*_*n*_ and variation of *α*, (**h**) emergence of the point of collapse computed in terms of supplier removal *f*_*n*_ and variation of *α*, and (**i**) emergence of the point of collapse computed in terms of global weight loss *f*_*w*_ and variation of *α*.

We subject both longitudinal networks and cross sectional networks to local and global perturbations. The resiliency patterns for all the networks using longitudinal data are shown in Supplementary Figures S16 to S24. The resiliency patterns for all the networks using cross sectional data are shown in Supplementary Figures S25 and S26. As expected, we observe that the networks formed using longitudinal data exhibit higher resiliency than the networks formed using cross sectional data. One can see that each realization generates a different point of collapse when subjected to perturbations in *f*_*n*_, *f*_*l*_, or *f*_*w*_ direction. However the emergence of the point of collapse (the first occurrence of the point of collapse does not change with respect to random perturbations. The exact point of collapse can be captured in the structure-parameter space. In $[{f}_{n}-\alpha ]$ space and $[{f}_{w}-\alpha ]$ space we are able to accurately predict the point of collapse (the stability analysis, derivation of the stable state, and computation of the point of collapse is explained in detail in Supplementary Information). So using the structure-parameter space one is able to predict the point of collapse in a space that involves local (*f*_*n*_) and global (*f*_*w*_) perturbations that occur outside the manufacturing facility and internal perturbations that is reflected in the parameter *α*. This is important since the aforementioned perturbations are measurable in real life. Figures 4 and 5 show the traceability of point of collapse in the structure-parameter space for networks created using longitudinal data and cross sectional data respectively. We observe that both internal perturbation and external perturbation deteriorate SM network resilience. Large internal perturbation shifts the point of collapse, where a small fraction of local perturbation (supplier or manufacturer removal) causes the system to collapse faster. Hence using the proposed approach we are able to reduce the multi-dimensions to manageable two dimensions that compute the effective throughput of manufacturers and suppliers in the SM network. In addition, we are able to accurately predict the point of collapse in a space that needs only the information about the underlying network structure and the internal reliability of the manufacturer and supplier facilities.

## Discussion

In this work we present an approach to measure the resiliency of a mutualistic supplier-manufacturer (SM) network. The proposed approach helps create a nonlinear dynamical model by integrating the network structure and the network parameters. The proposed model incorporates growth, internal reliability, price competition, technology competition and mutualistic interaction between the suppliers and manufacturers. The simplification approach helps reduce the multidimensional model to a manageable two-dimensional model, which is robust against noise and random parameter variations. Our results show that the resiliency of a SM network is a function of network structure and parameters. The resiliency is sensitive to structural properties of the network, namely, nestedness and density. Our model enables an accurate detection of emergence of the point of collapse as a measure of network resiliency which is highly predictable in the structure-parameter space. We observe that the SM network collapses in an accelerated fashion with the proportion of node or link removal and the reliability degrades resulting in decreased resiliency. For real-world applications, we observe that the global automotive industry is more resilient to local perturbations than to global perturbations, mainly due to high nestedness resulting from network design principles of redundancy adopted by the automotive industry. Global supply chains are becoming more integrated via cyber and physical interconnections. The emergence of Internet of Things (IOT) enables manufacturers and suppliers to measure system parameters in real time, given that current machines are equipped with advanced sensors for monitoring machine health. In addition, advanced statistical and machine learning models enable accurate prediction of machine failures. As time progresses, manufacturers and suppliers are able to obtain an accurate estimate of their reliability. The same cyber-physical principles help manufacturers and suppliers to obtain real time information regarding the network structure throughout the entire supply chain. Hence the current work is suitable for real world applications for predicting the collapse of SM networks. The proposed model can be adapted to any mutualistic SM networks. The proposed model fills an important existing theoretical gap in understanding and analyzing the resiliency of supply chains. We provide a comprehensive analytical framework from which further supply chain resiliency metrics can be developed.

Recovery patterns are generally time related behavior indicating the time to regain the original performance post a disruptive event^18^. The behavior of recovery patterns is influenced by factors such as speed of disruption onset, levels of disruptions and delays between intervention and performance response. As disruptions vary in severity and duration, so do the recovery patterns, which can be linear, concave, convex and non-specific. Hence combining all factors into a single metric for investigating SCR is challenging. Although recovery patterns were not covered within the scope of the current work, we believe this work will motivate and form a foundation for future research on recovery patterns of supply chains.

## Methods

### Real world SM networks

We use proprietary global automotive supply chain data obtained from Marklines database. The dataset has 376 manufacturers and 5229 suppliers who supply approximately 300 components. The data spans from 1999 to 2020. In the first version the dataset is divided into 21 sub-networks according to AA classification scheme as given in Supplementary Table S1. The classification scheme describes 21 categories where each category has multiple automotive components associated with it. These 21 sub-networks considers all the manufacturers and suppliers in each category across multiple years (longitudinal data). In such networks, manufacturers do not always get parts/products from the same suppliers every year. The network properties (densisty and nestedness) of these 21 sub-networks are given in Supplementary Table S2. In the second version of networks, we consider two automotive SM sub-networks just for the year 2017 (cross sectional data). The network properties (densisty and nestedness) are given in Supplementary Table S3.

#### Data source

https://www.marklines.com/portal_top_en.html.

### Computations

For testing the effectiveness of the dimension reduced model in presence of noise, Gaussian noise was added to the Eqs (1), (2), (3) and (4). The noise was added using the function awgn() in MATLAB computational software. For testing the effectiveness of the dimension reduced model in presence of random parameter variations, variations were introduced in form of uniform distribution using rand() function in MATLAB computational software. For all the synthetic networks and the real world SM networks, the network density, $\bar{a}$, of a bipartite network is computed as $\bar{a}=\frac{m}{I{\rm{\times }}J}$^49^, where *m* is the total number of interactions for all the nodes in the SM network; *I* is the number of manufacturers and *J* is the number of suppliers. The value of density varies from 0 to 1, 0 being low density and 1 being high density. The nestedness is computed using the NTC method^50^. The normalized value of nestedness varies form 0 to 1, 0 being low nestedness and 1 being high nestedness.

## Supplementary information

Supplementary Information

## Data Availability

The data for synthetic networks used in this work will be provided upon request.

## Code Availability

All the codes are generated using MATLAB computational software. Code will be made available upon request.
