# Supplementary material for: Resiliency of Mutualistic Supplier-Manufacturer Networks
Source: Sci Rep. 2019 Sep 19;9:13559. doi: 10.1038/s41598-019-49932-1 (PMC6753084; doi:10.1038/s41598-019-49932-1)
Supplement: Supplementary file 1 — Supplementary Information [file 41598_2019_49932_MOESM1_ESM.pdf]

Supplementary Information: Resiliency of Mutualistic Supplier-Manufacturer Networks

Mengkai Xu [a]
Srinivasan Radhakrishnan [a]
Sagar Kamarthi [a]
Xiaoning Jin [a,*]
a: Mechanical and Industrial Engineering, Northeastern University, Boston MA, USA
*: xi.jin@northeastern.edu

1. Nonlinear Dynamical Model of SM Mutualistic Network

Dynamics of a mutualistic system is represented by [1, 2, 3, 4]

$$\frac{dx_i(t)}{dt} = -x_i(d + sx_i) + \sum_{j=1}^{N} A_{ij}\gamma_{ij}x_i \frac{x_j^n}{\alpha^n + x_j^n} \tag{S1}$$

where x_i denotes the activity and $\frac{dx_i(t)}{dt}$ denotes the change in activity over time. In ecological applications, systems such as predator-prey, plant-pollinator to name a few, can be modeled in a bipartite network configuration where each class of bipartite is used to represent similar species (e.g., plant or pollinator) and the interactions between classes are represented as links. The dynamics of such mutualistic ecological systems are usually modeled using Equation S1, where x_i denotes the abundance of species i and $\frac{dx_i(t)}{dt}$ denotes the change in abundance over time. The abundance of a species is impacted negatively by the death rate d, and interspecies competition or environment carrying capacity s. The third term in Equation S1 captures the mutualistic interaction between the species that has a positive influence over the abundance of species x_i. Mutualism allows species to benefit because of their interconnectivity, i.e., the thriving of one species is mutually beneficial to the other species connected to it. One can observe that the dynamical equation is a sigmoidal type response function, that does not encourage unbounded growth. The environment carrying capacity limits the growth of individual species, and the mutualistic interactions also have a saturation effect where the abundance of one species does not linearly increase with the abundance of other species that it is connected to.

Similar to mutualistic ecological networks, we model the supplier-manufacturer (SM) network as a bipartite network with suppliers represented as one class and the manufacturers represented as the other class. The interactions between the suppliers and manufacturers are denoted by links. Studies have revealed a strong similarity between structural properties of mutualistic ecological networks and SM networks [5, 6, 7]. Network properties, mainly, nestedness, degree distribution, and modularity were significantly similar for ecological networks and SM networks [5]. One can emulate the dynamics of mutualistic ecological networks for SM networks. The dynamical equations governing the change in throughput of manufacturers and suppliers are as follows

$$\frac{dM_i}{dt} = \underbrace{M_i\left(1 - \frac{M_i}{K_i^{(M)}}\right)}_{\text{growth}} - \underbrace{\alpha_i^{(M)} M_i}_{\text{internal perturbation}} \underbrace{- M_i \sum_{j=1, j\neq i}^{Q_M} \beta_{ij}{}^{(M1)} M_j - M_i \sum_{j=1, j\neq i}^{Q_M} \beta_{ij}{}^{(M2)} M_j}_{\text{competition}} + \underbrace{M_i \frac{\sum_{k=1}^{Q_S} \gamma_{ik}{}^{(M)} S_k}{1 + h\sum_{k=1}^{Q_S} \gamma_{ik}{}^{(M)} S_k}}_{\text{mutualistic interaction}} + \mu_i{}^{(M)} \tag{S2}$$

$$\frac{dS_i}{dt} = S_i\left(1 - \frac{S_i}{K_i^{(S)}}\right) - \alpha_i^{(S)} S_i - S_i \sum_{j=1, j\neq i}^{Q_S} \beta_{ij}^{(S1)} S_j - S_i \sum_{j=1, j\neq i}^{Q_S} \beta_{ij}^{(S2)} S_j + S_i \frac{\sum_{k=1}^{Q_M} \gamma_{ik}^{(S)} M_k}{1 + h\sum_{k=1}^{Q_M} \gamma_{ik}^{(S)} M_k} + \mu_i^{(S)} \tag{S3}$$

The subscript M represents manufacturers and the subscript S represents suppliers. In Equation S2, M_i represents the throughput (production volume) of the manufacturer i. The first term represents the growth in throughput which is bounded (logistic growth) by the production capacity (K_i) of the manufacturing plant. Logistic growth models have extensively been investigated for ecological systems where the environment carrying capacity, and competition act as the growth limiting factors [8, 3]. Logistic growth behavior has been observed in new car registrations in Japan [9], car population in Italy [9], and annual production of natural resources [10, 11]. The second term represents internal perturbations which are captured by the internal reliability parameter α. The internal perturbation can manifest as machine shutdowns, labor unrest, quality issues and so on that have a negative impact on the throughput of a manufacturing plant. In the third term, parameter β, represents the price competition and the technology competition. The fourth term represents the mutualistic effect where interaction between manufacturers and suppliers have a positive effect on the throughput of a manufacturing plant. The fifth term μ indicates the production subcontracting/outsourcing intensity. In the long run, μ will have a negligible effect on a manufacturer's dynamics, but in the short term, μ exerts considerable influence. However, manufacturers and suppliers rarely outsource their proprietary components/products for the fear of creating unintended opportunities for future competition in reality. So even for the short term, one can assume a negligible effect of μ on the throughput of a SM network.

2. Solution in Absence of Mutualistic Interaction

When Equation S1 is used to model the dynamics of a mutualistic ecological system, the x_i is used to represent the abundance of the species i and $\frac{dx_i(t)}{dt}$ denotes the change in abundance over time. In such cases, the growth of a species is presented after incorporating in the mutualistic term. The absence of mutualistic interaction causes the abundance x_i to go to meagerness. The real world interpretation simply translates as the growth of a species without other interacting species. However, in the case of mutualistic SM networks, ideally, a manufacturing plant can produce if there is no extant resource shortage. In practice, manufacturers suffer from supply shortage in the absence of their respective suppliers. In the absences of inter-firm competition and mutualistic

interactions, namely the third and the fourth terms, Equations S2 and S3 can be written as follows

$$\frac{dM_i}{dt} = \underbrace{M_i\left(1 - \frac{M_i}{K_i^{(M)}}\right)}_{\text{growth}} - \underbrace{\alpha_i^{(M)} M_i}_{\text{internal perturbation}} \tag{S4}$$

$$\frac{dS_i}{dt} = S_i\left(1 - \frac{S_i}{K_i^{(S)}}\right) - \alpha_i^{(S)} S_i \tag{S5}$$

The solution for Equation S4 is obtained when $\frac{dM_i}{dt} = 0$.

$$M_i\left(1 - \frac{M_i}{K_i^{(M)}}\right) - \alpha_i^{(M)} M_i = 0 \tag{S6}$$

Hence, $M_i = 0$ or $1 - \frac{M_i}{K_i^{(M)}} - \alpha_i^{(M)} = 0$. Initially, when the plant utilization increases, the throughput of the plant increases exponentially. However, beyond a certain point, any increase in utilization is followed by a decrease in the throughput. The root cause for this trend is that the increase in utilization comes at the expense of avoiding equipment maintenance and complexity for production management which adversely affect the throughput. The production capacity $K_i^{(M)}$ indicates the maximum throughput of a manufacturing plant when operated under ideal conditions. When the manufacturers are starved of supply, the throughput of manufacturers collapse to zero ($M_i = 0$). Likewise, the absence of manufacturers constraints suppliers for demand, and the throughput of suppliers goes to zero ($S_i = 0$). Hence in the absence of mutualistic interaction, the feasible value of α is greater than 1.

3. Solution in Presence of Mutualistic Interaction

Mutualistic interactions between suppliers and manufacturers are captured by two dynamic Equations S2 and S3 where we consider a mutualistic supplier-manufacturer network consisting of Q_M manufacturers and Q_S suppliers. We assume the parameters to be independent of firm. The effective production capacity $K_i^{(M)} = K_i^{(S)} = K$; the reliability loss $\alpha_i^{(M)} = \alpha_i^{(S)} = \alpha$; the price competition parameter $\beta_{ij}^{(M1)} = \beta_{ij}^{(S1)} = \beta^{(1)}$ and technology competition parameter $\beta_{ij}^{(M2)} = \beta_{ij}^{(S2)} = \beta^{(2)}$, the outsourcing parameter $\mu_i^{(M)} = \mu_i^{(S)} = \mu$. The mutli-dimensional Equation S2 and S3 are reduced to two-dimensional equations given below

$$\begin{cases} \frac{dM_{eff}}{dt} = M_{eff}\left(1 - \frac{M_{eff}}{K}\right) - \alpha M_{eff} - \beta^{(M)}\left(M_{eff}\right)^2 + M_{eff}\frac{\langle\gamma_M\rangle S_{eff}}{1+h\langle\gamma_M\rangle S_{eff}} + \mu \\ \frac{dS_{eff}}{dt} = S_{eff}\left(1 - \frac{S_{eff}}{K}\right) - \alpha S_{eff} - \beta^{(S)}\left(S_{eff}\right)^2 + S_{eff}\frac{\langle\gamma_S\rangle M_{eff}}{1+h\langle\gamma_S\rangle M_{eff}} + \mu \end{cases} \tag{S7}$$

M_{eff} and P_{eff} are the effective throughput of the manufacturers and suppliers. The reduced model contains two differential dynamic equations: one for supplier and one for manufacturer. The dimension reduction procedures are given below.
The effective throughputs of manufacturer and supplier are used to indicate the average throughputs of the manufacturers and suppliers, the term incorporates throughput growth which is formulated as

$$M_i\left(1-\frac{M_i}{K_i^{(M)}}\right)\cong M_{eff}\left(1-\frac{M_{eff}}{K}\right) \quad \text{and} \quad S_i\left(1-\frac{S_i}{K_i^{(M)}}\right)\cong S_{eff}\left(1-\frac{S_{eff}}{K}\right) \tag{S8}$$

The internal perturbation (system reliability loss) is formulated as

$$\alpha_i^{(M)}M_i \cong \alpha M_{eff} \quad \text{and} \quad \alpha_i^{(S)}S_i \cong \alpha S_{eff} \tag{S9}$$

The interfirm competitions in terms of pricing and quality are integrated as

$$\begin{cases} M_i \sum_{j=1,j\neq i}^{Q_M} \beta_{ij}^{(M1)}M_j + M_i \sum_{j=1,j\neq i}^{Q_M} \beta_{ij}^{(M2)}M_j \cong \frac{\sum_{i=1}^{Q_M}\sum_{j=1,j\neq i}^{Q_M}\left(\beta^{(1)}+\beta^{(2)}\right)}{Q_M}\left(M_{eff}\right)^2 = \beta^{(M)}\left(M_{eff}\right)^2 \\ S_i \sum_{j=1,j\neq i}^{Q_S} \beta_{ij}^{(S1)}S_j + S_i \sum_{j=1,j\neq i}^{Q_S} \beta_{ij}^{(S2)}S_j \cong \frac{\sum_{i=1}^{Q_S}\sum_{j=1,j\neq i}^{Q_S}\left(\beta^{(1)}+\beta^{(2)}\right)}{Q_S}\left(S_{eff}\right)^2 = \beta^{(S)}\left(S_{eff}\right)^2 \end{cases} \tag{S10}$$

Finally, the mutualistic effects due to the network topology are averaged using the degree-weighted averaging method [2, 4] as shown below

$$\langle \gamma_M \rangle = \frac{\sum_{i=1}^{Q_M} \gamma_0 N_i^{1-\delta} N_i}{\sum_{i=1}^{Q_M} N_i} \quad \text{and} \quad \langle \gamma_S \rangle = \frac{\sum_{i=1}^{Q_S} \gamma_0 N_i^{1-\delta} N_i}{\sum_{i=1}^{Q_S} N_i} \tag{S11}$$

4. Stability Analysis

The reduced two-dimensional model is used to investigate the system stability. The stable state is obtained when $\frac{\mathrm{d}_{\mathrm{M_{eff}}}}{\mathrm{d}t} = 0$ and $\frac{\mathrm{d}_{\mathrm{M_{eff}}}}{\mathrm{d}t} = 0$. The aforementioned condition gives

$$\begin{cases} f_1\left(M_{eff}, S_{eff}\right) = \frac{dM_{eff}}{dt} = M_{eff}\left(1 - \frac{M_{eff}}{K}\right) - \alpha M_{eff} - \beta^{(M)}\left(M_{eff}\right)^2 + M_{eff}\frac{\langle \gamma_M \rangle S_{eff}}{1+h\langle \gamma_M \rangle S_{eff}} + \mu = 0 \\ f_2\left(M_{eff}, S_{eff}\right) = \frac{dS_{eff}}{dt} = S_{eff}\left(1 - \frac{S_{eff}}{K}\right) - \alpha S_{eff} - \beta^{(S)}\left(S_{eff}\right)^2 + S_{eff}\frac{\langle \gamma_S \rangle M_{eff}}{1+h\langle \gamma_S \rangle M_{eff}} + \mu = 0 \end{cases} \tag{S12}$$

To ensure the stability of transactions between suppliers and manufacturers, Equation S12 must satisfy

$$\begin{cases} \frac{df_1\left(M_{eff}, S_{eff}\right)}{dM_{eff}} = 1 - \frac{2M_{eff}}{K} - \alpha - 2\beta^{(M)}M_{eff} + \frac{\langle \gamma_M \rangle S_{eff}}{1+h\langle \gamma_M \rangle S_{eff}} < 0 \\ \frac{df_2\left(M_{eff}, S_{eff}\right)}{dS_{eff}} = 1 - \frac{2S_{eff}}{K} - \alpha - 2\beta^{(S)}S_{eff} + \frac{\langle \gamma_S \rangle M_{eff}}{1+h\langle \gamma_S \rangle M_{eff}} < 0 \end{cases} \tag{S13}$$

5. Stable State Analysis

Because $\mu^{(M)}$ and $\mu^{(S)}$ have negligible effects on system dynamics, $\mu^{(M)}$ and $\mu^{(S)}$ are approximated to zero. Hence Equation S12 can be written as

$$\begin{cases} f_1\left(M_{eff}, S_{eff}\right) \approx M_{eff}\left(1 - \frac{M_{eff}}{K}\right) - \alpha M_{eff} - \beta^{(M)}\left(M_{eff}\right)^2 + M_{eff}\frac{\langle \gamma_M \rangle S_{eff}}{1+h\langle \gamma_M \rangle S_{eff}} = 0 \\ f_2\left(M_{eff}, S_{eff}\right) \approx S_{eff}\left(1 - \frac{S_{eff}}{K}\right) - \alpha S_{eff} - \beta^{(S)}\left(S_{eff}\right)^2 + S_{eff}\frac{\langle \gamma_S \rangle M_{eff}}{1+h\langle \gamma_S \rangle M_{eff}} = 0 \end{cases} \tag{S14}$$

$M'_{eff} = 0$ and $S'_{eff} = 0$ are one of the roots for Equation S14. The other roots can be derived as follows

$$\begin{cases} \left(1 - \frac{M_{eff}}{K}\right) - \alpha - \beta^{(M)}M_{eff} + \frac{\langle \gamma_M \rangle S_{eff}}{1+h\langle \gamma_M \rangle S_{eff}} = 0 \\ \left(1 - \frac{S_{eff}}{K}\right) - \alpha - \beta^{(S)}S_{eff} + \frac{\langle \gamma_S \rangle M_{eff}}{1+h\langle \gamma_S \rangle M_{eff}} = 0 \end{cases} \tag{S15}$$

Equation S15 can be reformulated as

$$
\begin{aligned}
g_1\left(M_{eff}\right) = -h\left(1+K\beta^{(M)}\right)\langle\gamma_S\rangle\left[\left(1+K\beta^{(S)}\right)+K\langle\gamma_M\rangle+hK(1-\alpha)\langle\gamma_M\rangle\right]{M_{eff}}^2 \\
+\left[Kh(1-\alpha)\left(\langle\gamma_S\rangle\left(1+K\beta^{(S)}\right)+\langle\gamma_M\rangle\left(1+K\beta^{(M)}\right)\right)\right. \\
-\left(1+K\beta^{(M)}\right)\left(1+K\beta^{(S)}\right)+2K^2h(1-\alpha)\langle\gamma_S\rangle\langle\gamma_M\rangle \\
\left.+K^2h^2(1-\alpha)^2\langle\gamma_S\rangle\langle\gamma_M\rangle+K^2\langle\gamma_S\rangle\langle\gamma_M\rangle\right] \\
M_{eff}+\left[K(1-\alpha)\left(1+K\beta^{(S)}+K\langle\gamma_M\rangle\right)\right. \\
\left.+Kh(1-\alpha)^2\langle\gamma_M\rangle\right] \\
=0 \\
g_2\left(S_{eff}\right) = -h\left(1+K\beta^{(S)}\right)\langle\gamma_M\rangle\left[\left(1+K\beta^{(M)}\right)+K\langle\gamma_S\rangle+hK(1-\alpha)\langle\gamma_S\rangle\right]{S_{eff}}^2 \\
+\left[Kh(1-\alpha)\left(\langle\gamma_M\rangle\left(1+K\beta^{(M)}\right)+\langle\gamma_S\rangle\left(1+K\beta^{(S)}\right)\right)\right. \\
-\left(1+K\beta^{(S)}\right)\left(1+K\beta^{(M)}\right)+2K^2h(1-\alpha)\langle\gamma_S\rangle\langle\gamma_M\rangle \\
\left.+K^2h^2(1-\alpha)^2\langle\gamma_S\rangle\langle\gamma_M\rangle+K^2\langle\gamma_S\rangle\langle\gamma_M\rangle\right] \\
S_{eff}+\left[K(1-\alpha)\left(1+K\beta^{(M)}+K\langle\gamma_S\rangle\right)\right. \\
\left.+Kh(1-\alpha)^2\langle\gamma_S\rangle\right] \\
=0
\end{aligned}
\tag{S16}
$$

The product of $g_1(M_{eff})$ and M_{eff} and product of $g_2(S_{eff})$ and S_{eff} in Equation S14 can be expressed as

$$f_1\left(M_{eff}, S_{eff}\right) \approx M_{eff} g_1\left(M_{eff}\right) = 0 \quad \text{and} \quad f_2\left(M_{eff}, S_{eff}\right) \approx S_{eff} g_2\left(M_{eff}\right) = 0 \tag{S17}$$

6. Graphical Solution

Equation S17 can be solved graphically as shown in Fig. S1.
Let,
$A_1 = -h(1 + K\beta^{(M)}) < \gamma_S > [(1 + K\beta^{(S)}) + K < \gamma_M > +hK(1 - \alpha) < \gamma_M >]$
$B_1 = Kh(1-\alpha)[< \gamma_S > (1+K\beta^{(S)})+ < \gamma_M > (1+K\beta^{(M)})] - (1+K\beta^{(S)})(1+K\beta^{(M)}) + 2K^2h(1-\alpha) < \gamma_M >< \gamma_S > +K^2h^2(1 - \alpha)^2 < \gamma_M >< \gamma_S > +K^2 < \gamma_M >< \gamma_S >$
$C_1 = K(1 - \alpha)(1 + K\beta^{(S)} + K < \gamma_M >) + Kh(1 - \alpha)^2 < \gamma_M >$
$A_2 = -h(1 + K\beta^{(S)}) < \gamma_M > [(1 + K\beta^{(M)}) + K < \gamma_S > +hK(1 - \alpha) < \gamma_S >]$
$B_2 = Kh(1-\alpha)[< \gamma_M > (1+K\beta^{(M)})+ < \gamma_S > (1+K\beta^{(S)})] - (1+K\beta^{(M)})(1+K\beta^{(S)}) + 2K^2h(1-\alpha) < \gamma_M >< \gamma_S > +K^2h^2(1 - \alpha)^2 < \gamma_M >< \gamma_S > +K^2 < \gamma_M >< \gamma_S >$
$C3 = K(1 - \alpha)(1 + K\beta^{(M)} + K < \gamma_S >) + Kh(1 - \alpha)^2 < \gamma_S >$
We assume that the initial physical states for both manufacturers and suppliers are high. The stable-state of the manufacturer is determined by $B_1^2 - 4A_1C_1$. When $B_1^2 - 4A_1C_1 > 0$, the stable-state of manufacturer is in high throughput state M^H (see Figure S1a red color line). When $B_1^2 - 4A_1C_1 = 0$, the only stable-state of manufacturer is in low throughput state M^L (see Fig. S1a blue color line). When $B_1^2 - 4A_1C_1 < 0$, the only stable-state of manufacturer is in low throughput state M^L (see Fig. S1a green color line). Likewise, same mechanism works for the suppliers. The stable-state of the supplier is determined by $B_2^2 - 4A_2C_2$. When $B_2^2 - 4A_2C_2 > 0$, the stable-state of supplier is in high throughput state S^H (see Fig. S1b red color line). When $B_2^2 - 4A_2C_2 = 0$, the only stable-state of supplier is in low throughput state S^L (see Fig. S1b blue color line). When $B_2^2 - 4A_2C_2 < 0$, the only stable-state of manufacturer is in low throughput state S^L (see Fig. S1b green color line).
It is physically complex to solve Equation S17. We set the following parameter values to solve Equation S17:
$K_i = K = 1$, $\alpha_i = \alpha = 1.2$, $\beta_{ij}(1) = \beta(1) = 0.0001$, $\beta_{ij}(2) = \beta(2) = 0.0001$, $\mu_i = \mu = 0.0001$, $h = 0.5$
We use the synthetic network (high nestedness, low density) as one example to illustrate the derivation of the roots. $\beta(M) = 0.0018$ and $\beta(S) = 0.005$ are obtained from Equation S5. $< \gamma_S >= 2.1751$ and $< \gamma_M >= 3.6399$ are calculated using Equation S6. Since $B_1^2 - 4A_1C_1 > 0$ and $B_2^2 - 4A_2C_2 > 0$, we obtain $M_L = 0$, $M_M = 0.182$ and $M_H = 1.008$. Since the initial physical state is high, $M_H = 1.008$ is the stable-state solution. Likewise, the steady-state solution for the supplier is $S_H = 0.8419$ ($S_L = 0$, $S_M = 0.1299$).

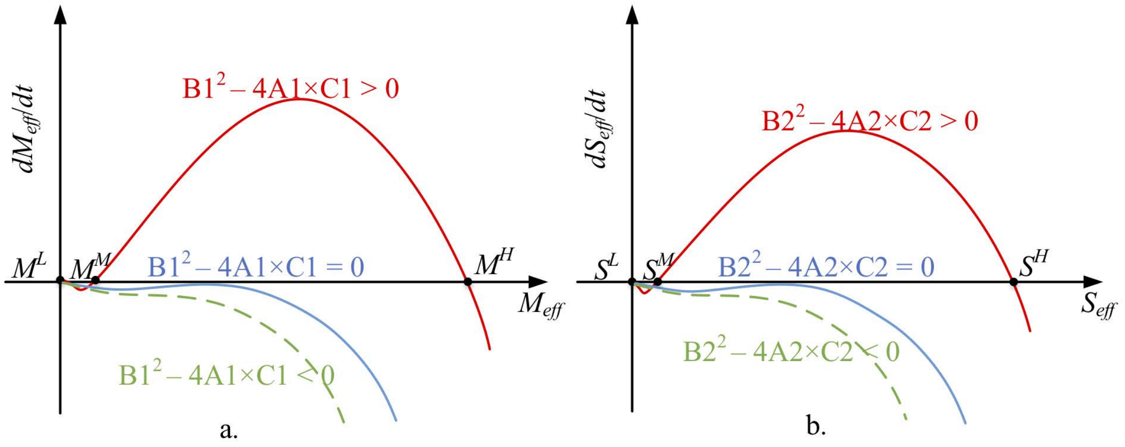

Figure S1: Graphical computation of solution for Equation S17 . Here the initial physical state of the system is high $M_{eff} = 5, S_{eff} = 5$. (a) The red curves correspond to $f_1(M_{eff}, S_{eff})$ term in Equation S12. When $B_1^2 - 4A_1C_1 > 0, f_1(M_{eff}, S_{eff}) = 0$ ($\frac{dM_{eff}}{dt} = 0$) has three roots M^L, M^M and M^H. Given the conditions (Equation S13) for ensuring stability, roots M^L and M^H are the stable fixed points. M^M is the unstable fixed point where a disturbance drives the system to approach M^L and leads to a complete loss in throughput. The blue curve corresponds to the scenario $B_1^2 - 4A_1C_1 = 0$ when, $f_1(M_{eff}, S_{eff})$ has two roots M^L and M^M. The only stable fixed point is M^L. The green dashed curve corresponds to the scenario $B_1^2 - 4A_1C_1 < 0$ when, $f_1(M_{eff}, S_{eff})$ has only one stable fixed point M^L. When $B_1^2 - 4A_1C_1 \leq 0$, the SM system is unable to recover from a perturbation thus rendering a non-resilient system. (b) The red curves corresponds to $f_2(M_{eff}, S_{eff}) = 0$ ($\frac{dS_{eff}}{dt} = 0$) in Equation S17. When $B_2^2 - 4A_2C_2 > 0, f_2(M_{eff}, S_{eff})$ has three roots S^L, S^M and S^H. Given the conditions (Equation S13) for ensuring stability, roots S^L and S^H are the stable fixed points. S^M is the unstable fixed point where a disturbance drives the system to approach S^L and leads to a complete loss in throughput. The blue curve corresponds to the scenario $B_2^2 - 4A_2C_2 = 0$ when, $f_2(M_{eff}, S_{eff})$ has two roots S^L and S^M. The only stable fixed point is S^L. The green dashed curve corresponds to the scenario $B_1^2 - 4A_1C_1 < 0$ when, $f_2(M_{eff}, S_{eff})$ has only one stable fixed point S^L. When $B_2^2 - 4A_2C_2 \leq 0$, the SM system is unable to recover from a perturbation thus rendering a non-resilient system

.

7. Determining the Point of Collapse

System at the point of collapse delivers a zero throughput and is irrecoverable. We use Fig. S1 to illustrate the emergence of point of collapse considering manufacturer's and supplier's dynamics and network structure. The emergence of point of collapse happens when $B_1^2 - 4A_1C_1 \leq 0$ as shown in Fig. S1a (blue and green color lines) or $B_2^2 - 4A_2C_2 \leq 0$ as shown in Fig. S1b (blue and green color lines). Since $B_1^2 - 4A_1C_1$ and $B_2^2 - 4A_2C_2$ will not approach zero simultaneously.

In addition to $B_1^2 - 4A_1C_1$ and $B_2^2 - 4A_2C_2$, insight for the stable state analysis around the point of collapse is offered by the mutualistic interaction term. Assume that $B_1^2 - 4A_1C_1$ approaches zero or has a negative value and $B_2^2 - 4A_2C_2$ is still positive, we observe $M_{eff} = 0$ (manufacturer throughput goes to zero when $B_1^2 - 4A_1C_1 \leq 0$) and the mutualistic interaction term for the suppliers becomes zero accordingly. Thus S_{eff} becomes zero (mutualistic interaction has positive contribution to the throughput of suppliers). The reverse works the same for the manufacturers. Thus, irrespective of terms $B_1^2 - 4A_1C_1$ ($B_2^2 - 4A_2C_2 > 0$) or $B_2^2 - 4A_2C_2$ ($B_1^2 - 4A_1C_1 < 0$) approach zero first, the SM system collapses when $B_1^2 - 4A_1C_1 \leq 0$ or $B_2^2 - 4A_2C_2 \leq 0$.

The point of collapse can be computed and visualized in a space that combines both parameter and network structure information. As explained in the main section of the paper, irrespective of the production capacity, a 100 percent capacity utilization reduces the plant throughput. For this reason, the ideal throughput of the plant can be set at 100 percent i.e., $K = 1$. For the short term, the competition parameter can be set to $\beta^{(1)} = \beta^{(2)} = 0.0001$. Thus, $B_1^2 - 4A_1C_1$ is decided by the internal perturbation α and topological structure $< \gamma_S >$ and $< \gamma_M >$. We use the synthetic network (high nestedness, low density) to demonstrate the emergence of collapse point in functions of α and f_n or f_l. f_n or f_l captures the topological information $< \gamma S >$ and $< \gamma M >$ (see Fig. S2).

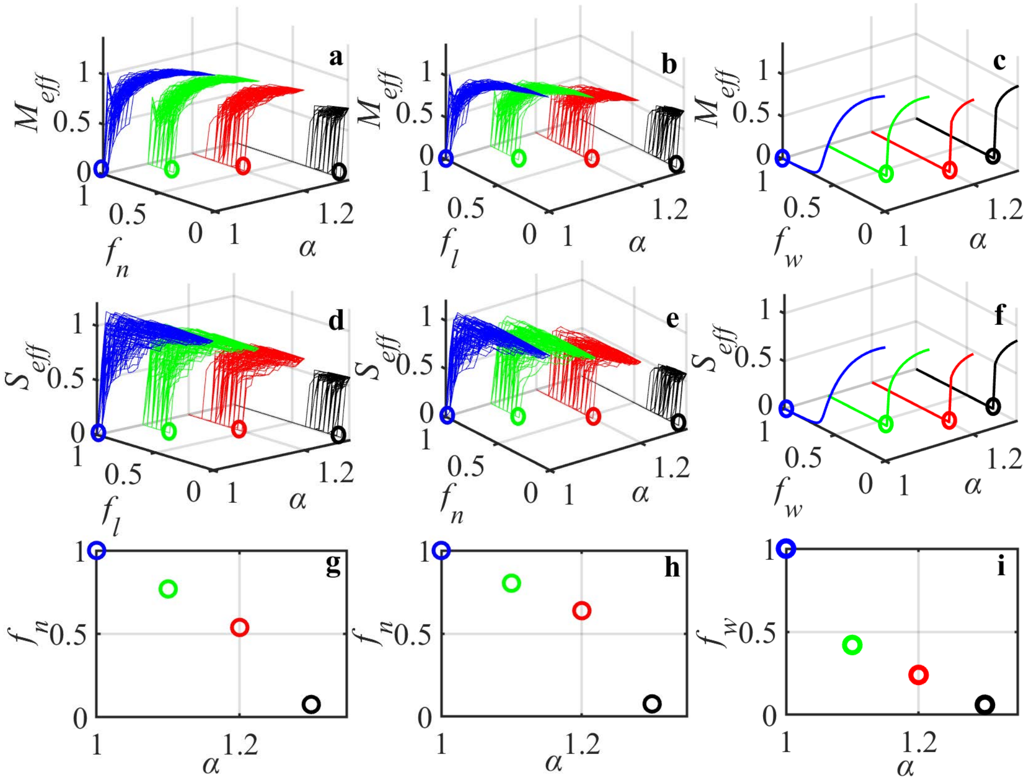

Figure S2: Emergence of point of collapse for SM network. The SM network has 10 manufacturers and 26 suppliers (a) Resilience function M_{eff} vs. manufacturer removal f_n with the parameter regime of α, (b) Resilience function M_{eff} vs. manufacturer link loss f_l with the parameter regime of α, (c) Resilience function M_{eff} vs. global weight loss f_w with the parameter regime of α, (d) Resilience function S_{eff} vs. supplier link loss f_l with the parameter regime of α, (e) Resilience function S_{eff} vs. supplier removal f_n with the parameter regime of α, (f) Resilience function S_{eff} vs. global weight loss f_w with the parameter regime of α, (g) Emergence of the point of collapse computed in terms of manufacturer removal f_n and variation of α, (h) Emergence of the point of collapse computed in terms of supplier removal f_n and variation of α, (i) Emergence of the point of collapse computed in terms of global weight loss f_n and variation of α .

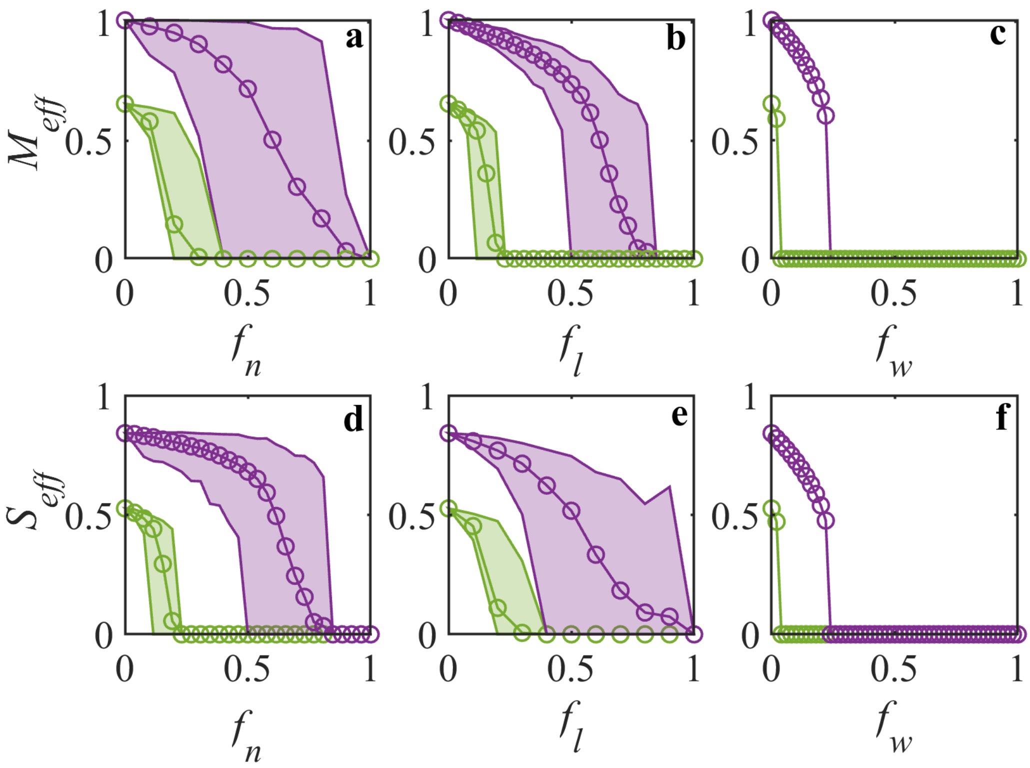

Figure S3: Effects of local and global perturbations on SM network with 10 manufacturers and 26 suppliers. Violet represents high nested network structure (0.95), and green represents low nested network structure (0.19). The line with circle is the average of 100 realizations and the shaded area shows the lower bound and the upper bound values. The network density for both the high and low nested networks is maintained constant at 0.30. (a) and (d) show the effective throughput for manufacturer and supplier when subjected to manufacturer removal f_n and supplier removal f_n respectively. (b) and (e) show the effective throughput for manufacturer and supplier when subjected to supplier removal f_l and manufacturer removal f_l respectively. (c) and (f) represent the effect of global perturbation on effective throughput of manufacturer and supplier respectively in the form of weight reduction f_w. $K = 1$, $\alpha = 1.2$, $\beta = 0.0001$, $\gamma_0 = 1$, $\delta = 0.5$, $h = 0.5$.

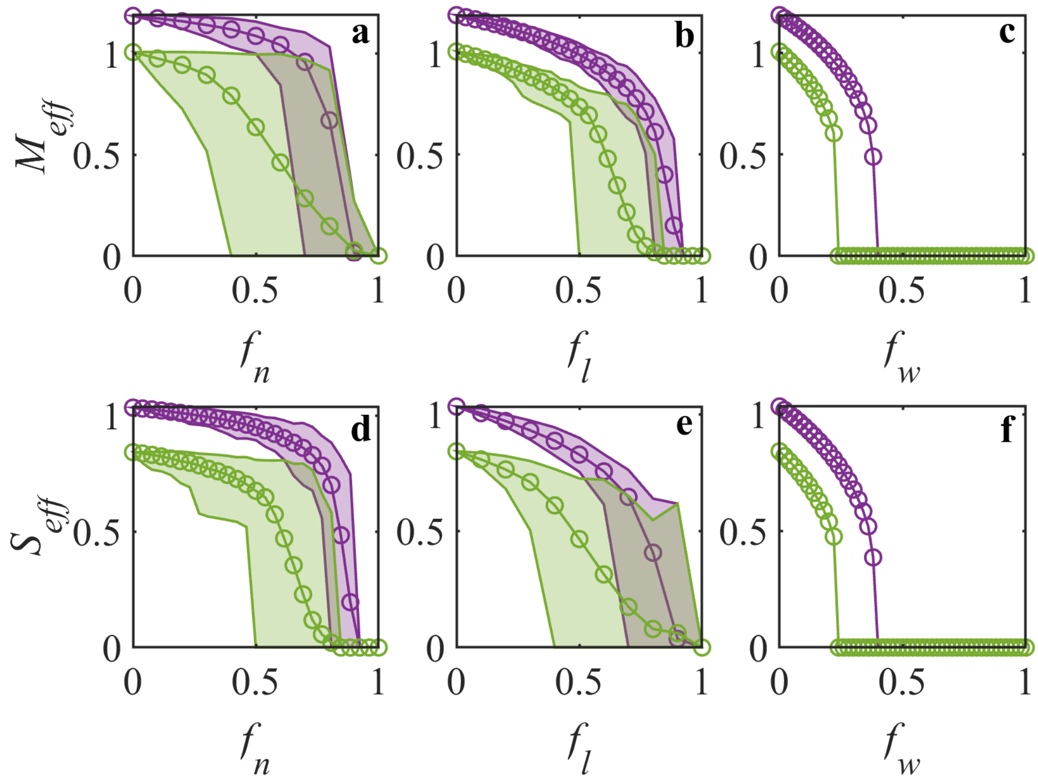

Figure S4: Effects of local and global perturbations on SM network with 10 manufacturers and 26 suppliers. Violet represents high-density network structure (0.68), and green represents low-density network structure (0.30). The line with circle is the average of 100 realizations and the shaded area shows the lower bound and the upper bound values. The nestedness for both the high- and low-density networks is maintained constant at 0.95. (a) and (d) show the effective throughput for manufacturer and supplier when subjected to manufacturer removal f_n and supplier removal f_n respectively. (b) and (e) show the effective throughput for manufacturer and supplier when subjected to supplier removal f_l and manufacturer removal f_l respectively. (c) and (f) represent the effect of global perturbation on effective throughput of manufacturer and supplier in the form of weight reduction f_w. $K = 1$, $\alpha = 1.2$, $\beta = 0.0001$, $\gamma_0 = 1$, $\delta = 0.5$, $h = 0.5$.

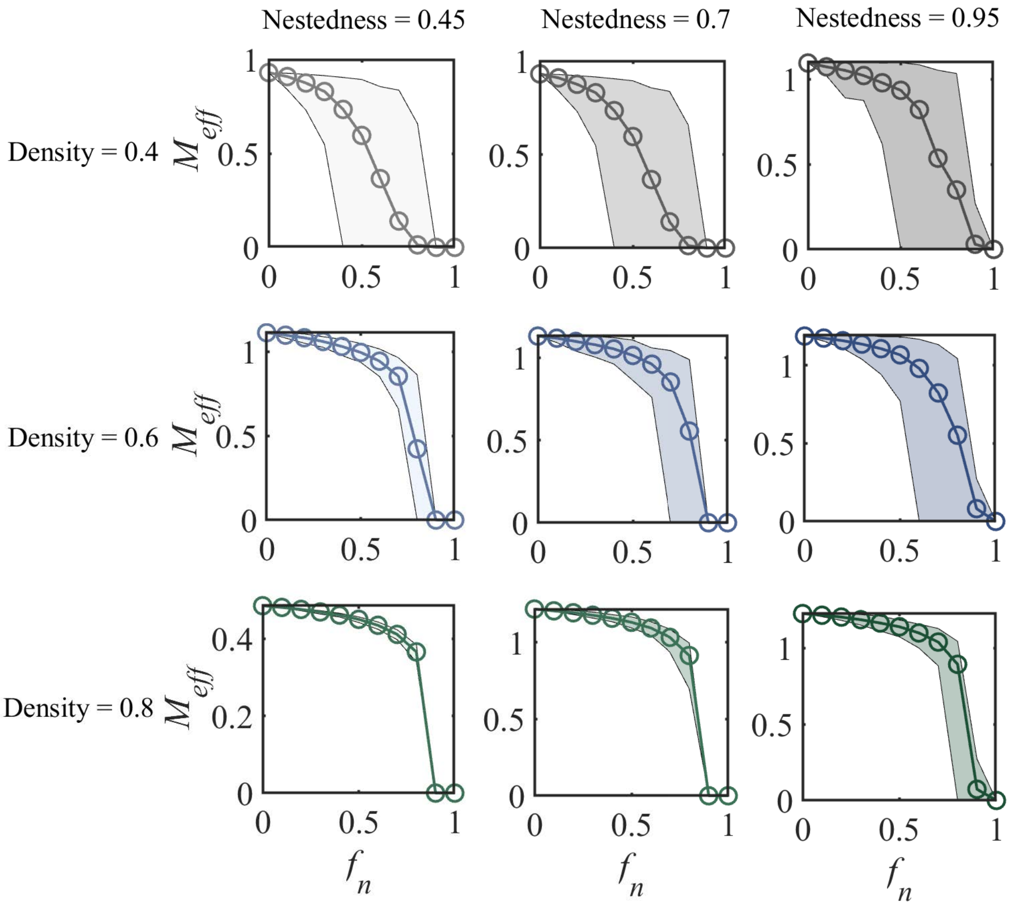

Figure S5: Resilience profile of simulated SM network under varying degree of nestedness and network density. All the figures show the effective throughput of the manufacturers when subjected to manufacturer removal (f_n). The line with circle is the average of 100 realizations and the shaded area shows the lower bound and the upper bound values. $K = 1, \beta = 0.0001, \gamma_0 = 1, \delta = 0.5, h = 0.5$. The simulated network has 10 manufacturers and 26 suppliers.

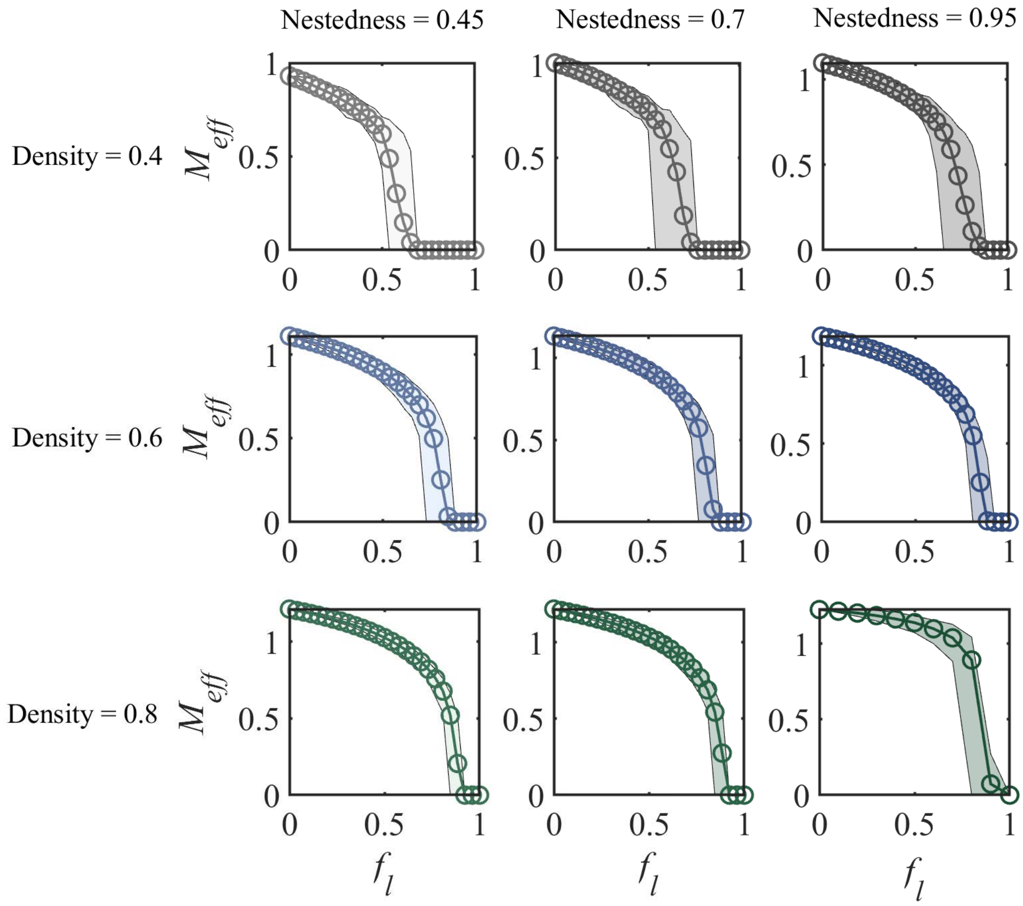

Figure S6: Resilience profile of simulated SM network under varying degree of nestedness and network density. All the figures show the effective throughput of the manufacturers when subjected to supplier removal (f_l). The line with circle is the average of 100 realizations and the shaded area shows the lower bound and the upper bound values. $K = 1$, $\beta = 0.0001$, $\gamma_0 = 1$, $\delta = 0.5$, $h = 0.5$.

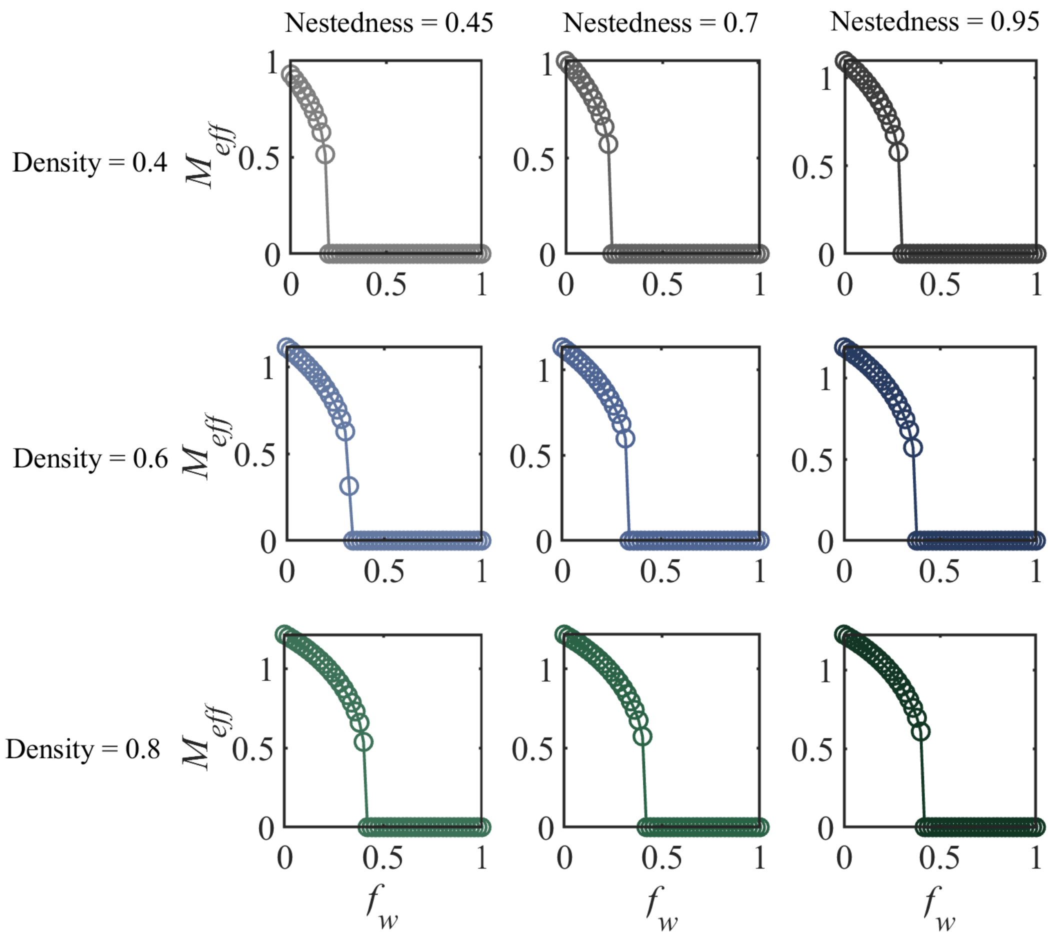

Figure S7: Resilience profile of simulated SM network under varying degree of nestedness and network density. All the figures show the effective throughput of the manufacturers when subjected to weight reduction (f_w). The line with circle is the average of 100 realizations and the shaded area shows the lower bound and the upper bound values. $K = 1$, $\beta = 0.0001$, $\gamma_0 = 1$, $\delta = 0.5$, $h = 0.5$. The simulated network has 10 manufacturers and 26 suppliers.

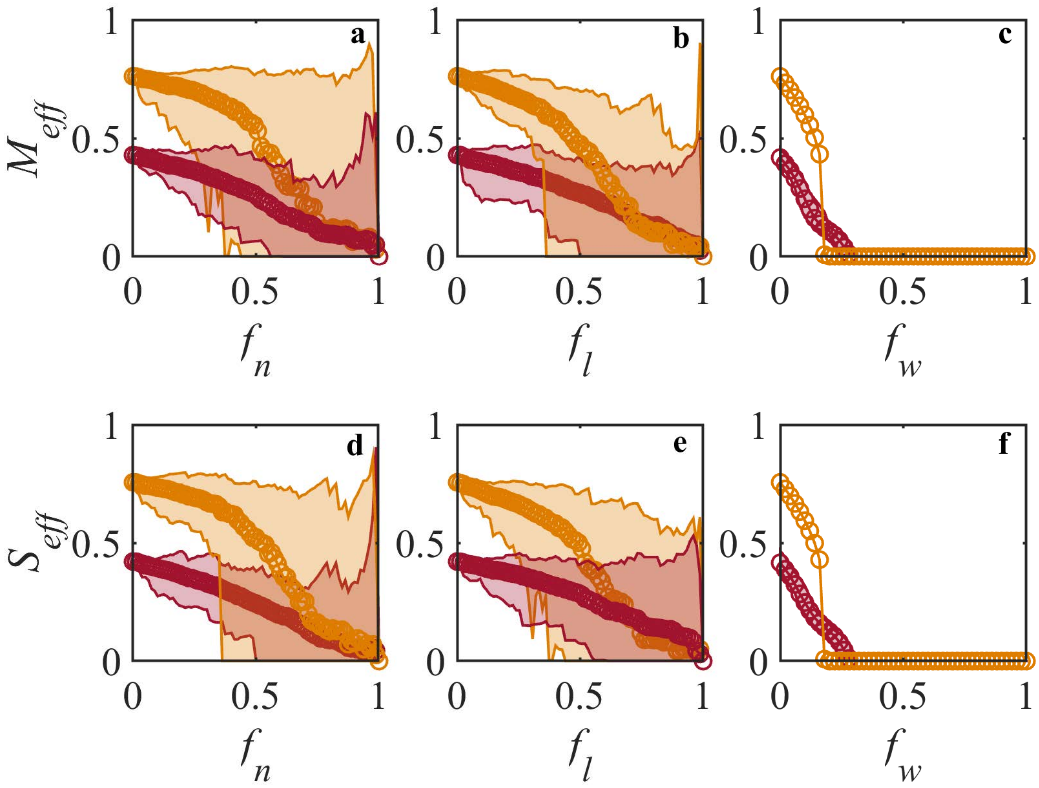

Figure S8: Robustness of dimension reduced model against noise. (a) and (d) show the throughput for manufacturer and supplier when subjected to manufacturer removal f_n and supplier removal f_n respectively. (b) and (e) show the throughput for manufacturer and supplier when subjected to supplier removal f_l and manufacturer removal f_l respectively. (c) and (f) represent the effect of global perturbation on throughput of manufacturer and supplier respectively in the form of weight reduction f_w. Maroon color represents multidimensional model (Equations 1 and 2 from the main text), Orange color represents dimension reduced model (Equations 4 and 5 from the main text). The line with circle is the average of 100 realizations and the shaded area shows the lower bound and the upper bound values. $K = 1$, $\alpha = 1.2$, $\beta = 0.0001$, $\gamma_0 = 1$, $\delta = 0.5$, $h = 0.5$. A Gaussian noise of strength 0.1 was added to both the multidimensional model and the reduced model. The network considered here is for suppliers who supplied drive train components to the manufacturers for the year 2017. It has 75 manufacturers and 80 suppliers. The nestedness value is 0.9703 and the density value is 0.0323.

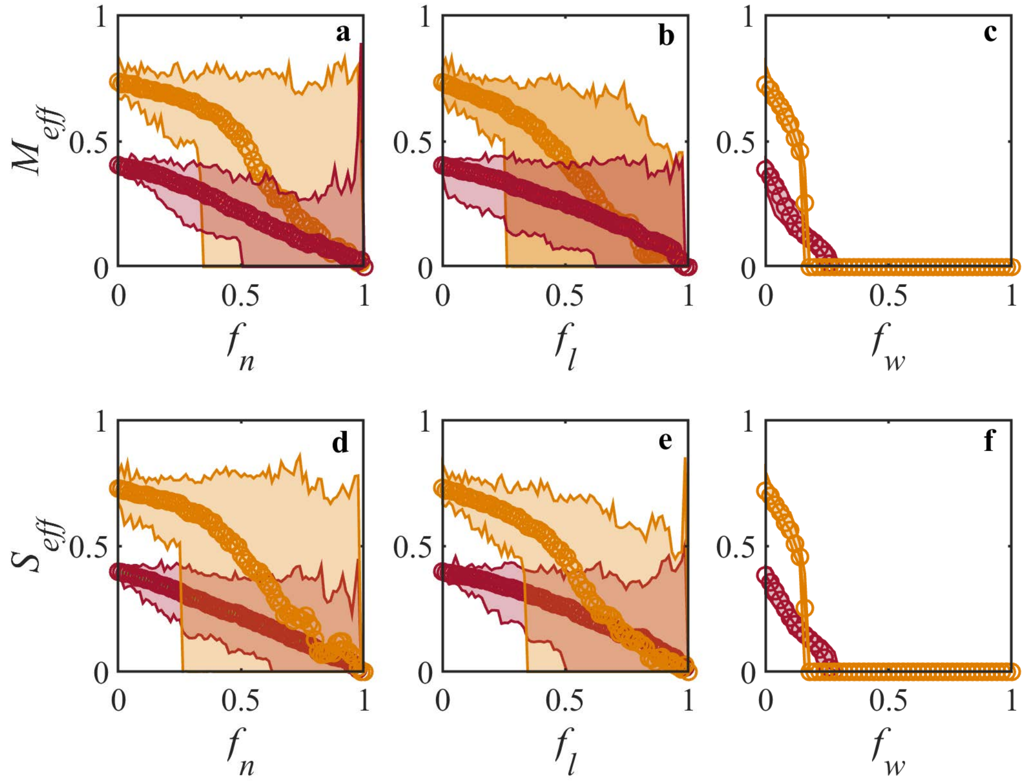

Figure S9: Robustness of dimension reduced model against random parameter variation of K. (a) and (d) show the throughput for manufacturer and supplier when subjected to manufacturer removal f_n and supplier removal f_n respectively. (b) and (e) show the throughput for manufacturer and supplier when subjected to supplier removal f_l and manufacturer removal f_l respectively. (c) and (f) represent the effect of global perturbation on throughput of manufacturer and supplier respectively in the form of weight reduction f_w. Maroon color represents multidimensional model (Equations 1 and 2 from the main text), Orange color represents dimension reduced model (Equations 4 and 5 from the main text). The line with circle is the average of 100 realizations and the shaded area shows the lower bound and the upper bound values. $\alpha = 1.2$, $\beta = 0.0001$, $\gamma_0 = 1$, $\delta = 0.5$, $h = 0.5$. The values of parameter K was subjected to random variations using an uniform distribution $U[0.8, 1.2]$. The network considered here is for suppliers who supplied drive train components to the manufacturers for the year 2017. It has 75 manufacturers and 80 suppliers. The nestedness value is 0.9703 and the density value is 0.0323.

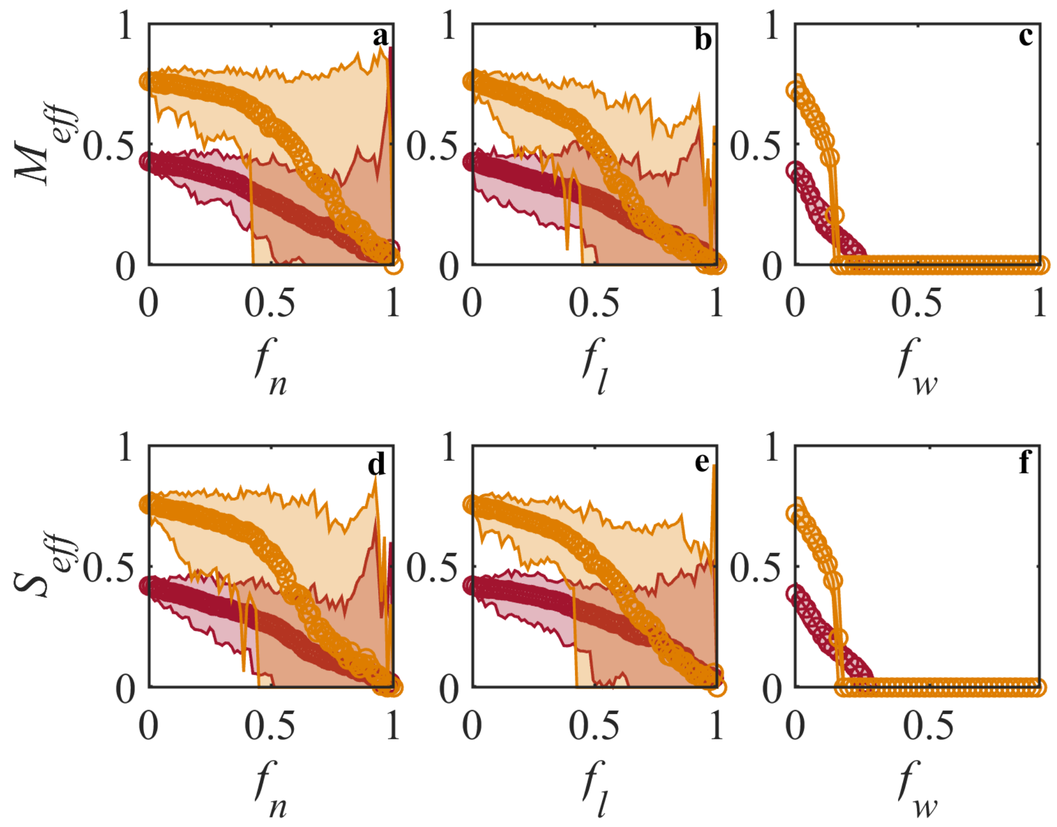

Figure S10: Robustness of dimension reduced model against random parameter variation of α. (a) and (d) show the throughput for manufacturer and supplier when subjected to manufacturer removal and supplier removal respectively. (b) and (e) show the throughput for manufacturer and supplier when subjected to supplier removal and manufacturer removal respectively. (c) and (f) represent the effect of global perturbation on throughput of manufacturer and supplier respectively in form of weight reduction. Maroon color represents multidimensional model (Equations 1 and 2 from the main text), Orange color represents dimension reduced model (Equations 4 and 5 from the main text). The line with circle is the average of 100 realizations and the shaded area shows the lower bound and the upper bound values. $K = 1$, $\beta = 0.0001$, $\gamma_0 = 1$, $\delta = 0.5$, $h = 0.5$. The value of parameter α was subjected to random variations using a uniform distribution $U[1, 1.2]$. The network considered here is for suppliers who supplied drive train components to the manufacturers for the year 2017. It has 75 manufacturers and 80 suppliers. The nestedness value is 0.9703 and the density value is 0.0323.

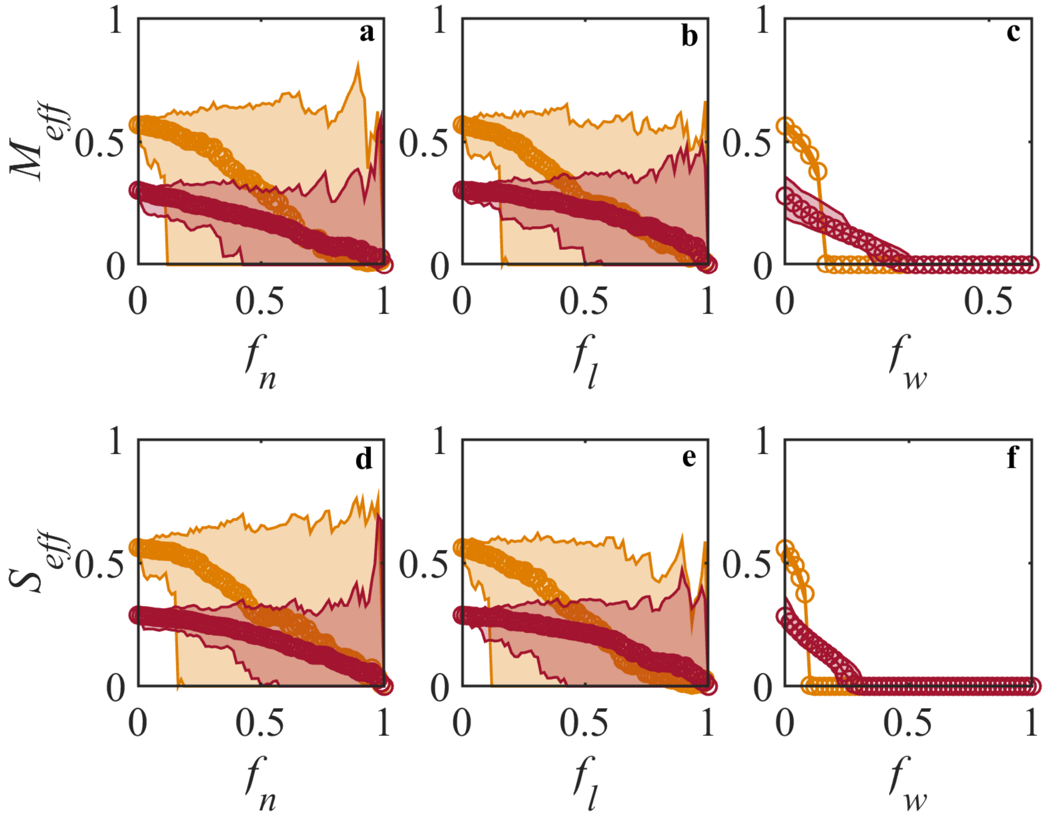

Figure S11: Robustness of dimension reduced model against random parameter variation of β. (a) and (d) show the throughput for manufacturer and supplier when subjected to manufacturer removal and supplier removal respectively. (b) and (e) show the throughput for manufacturer and supplier when subjected to supplier removal and manufacturer removal respectively. (c) and (f) represent the effect of global perturbation on throughput of manufacturer and supplier respectively in form of weight reduction. Maroon color represents multidimensional model (Equations 1 and 2 from the main text), Orange color represents dimension reduced model (Equations 4 and 5 from the main text). The line with circle is the average of 100 realizations and the shaded area shows the lower bound and the upper bound values. $K = 1$, $\alpha = 1.2$, $\gamma_0 = 1$, $\delta = 0.5$, $h = 0.5$. The values of parameter β were subjected to random variations using an uniform distribution $U[0.001, 0.002]$. The network considered here is for suppliers who supplied drive train components to the manufacturers for the year 2017. It has 75 manufacturers and 80 suppliers. The nestedness value is 0.9703 and the density value is 0.0323.

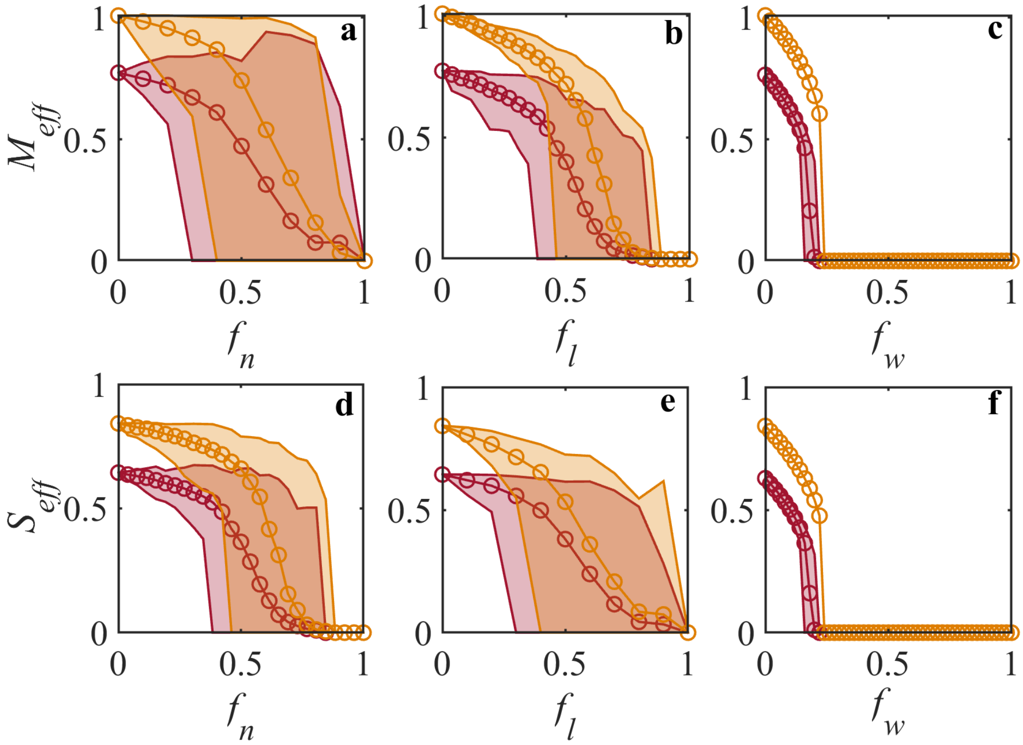

Figure S12: Robustness of dimension reduced model against noise. (a) and (d) show the throughput for manufacturer and supplier when subjected to manufacturer removal f_n and supplier removal f_n respectively. (b) and (e) show the throughput for manufacturer and supplier when subjected to supplier removal f_l and manufacturer removal f_l respectively. (c) and (f) represent the effect of global perturbation on throughput of manufacturer and supplier respectively in the form of weight reduction f_w. Maroon color represents multidimensional model (Equations 1 and 2 from the main text), Orange color represents dimension reduced model (Equations 4 and 5 from the main text). The line with circle is the average of 100 realizations and the shaded area shows the lower bound and the upper bound values. $K = 1$, $\alpha = 1.2$, $\beta = 0.0001$, $\gamma_0 = 1$, $\delta = 0.5$, $h = 0.5$. A Gaussian noise of strength 0.1 was added to both the multidimensional model and the reduced model. The synthetic network includes 10 manufacturers and 26 suppliers.

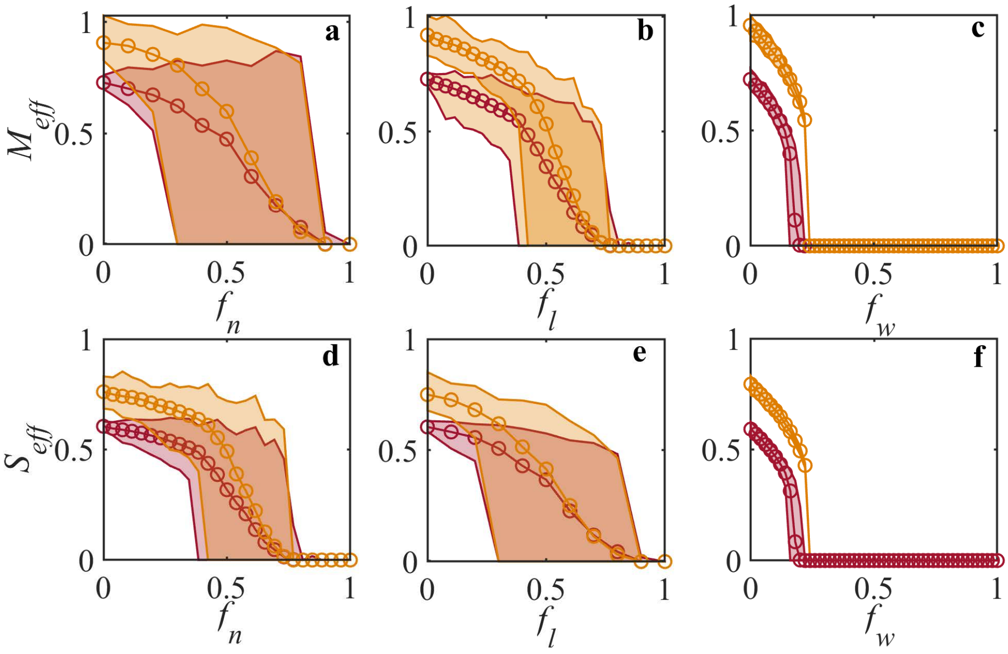

Figure S13: Robustness of dimension reduced model against random parameter variation of K. (a) and (d) show the throughput for manufacturer and supplier when subjected to manufacturer removal f_n and supplier removal f_n respectively. (b) and (e) show the throughput for manufacturer and supplier when subjected to supplier removal f_l and manufacturer removal f_l respectively. (c) and (f) represent the effect of global perturbation on throughput of manufacturer and supplier respectively in form of weight reduction f_w. Maroon color represents multidimensional model (Equations 1 and 2 from the main text), Orange color represents dimension reduced model (Equations 4 and 5 from the main text). The line with circle is the average of 100 realizations and the shaded area shows the lower bound and the upper bound values. $\alpha = 1.2$, $\beta = 0.0001$, $\gamma_0 = 1$, $\delta = 0.5$, $h = 0.5$. The value of parameter K was subjected to random variations using a uniform distribution $U[0.8, 1.2]$. The synthetic network includes 10 manufacturers and 26 suppliers.

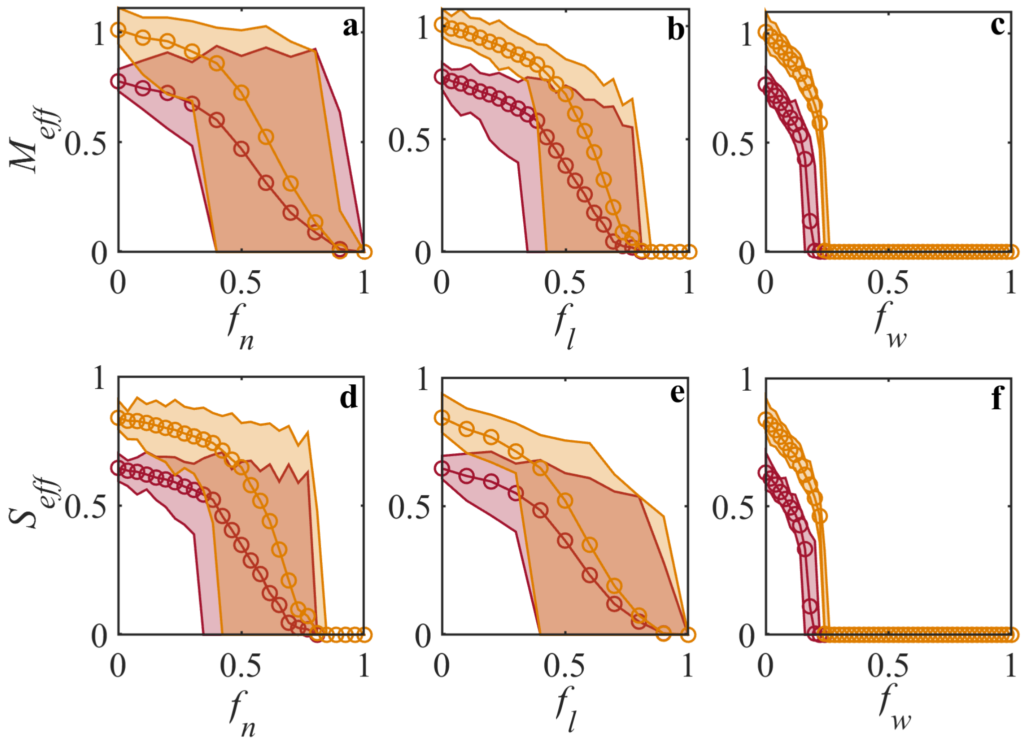

Figure S14: Robustness of dimension reduced model against random parameter variation of α. (a) and (d) show the throughput for manufacturer and supplier when subjected to manufacturer removal and supplier removal respectively. (b) and (e) show the throughput for manufacturer and supplier when subjected to supplier removal and manufacturer removal respectively. (c) and (f) represent the effect of global perturbation on throughput of manufacturer and supplier respectively in form of weight reduction. Maroon color represents multidimensional model (Equations 1 and 2 from the main text), Orange color represents dimension reduced model (Equations 4 and 5 from the main text). The line with circle is the average of 100 realizations and the shaded area shows the lower bound and the upper bound values. $K = 1$, $\beta = 0.0001$, $\gamma_0 = 1$, $\delta = 0.5$, $h = 0.5$. The value of parameter α was subjected to random variations using a uniform distribution $U[1, 1.4]$. The synthetic network includes 10 manufacturers and 26 suppliers.

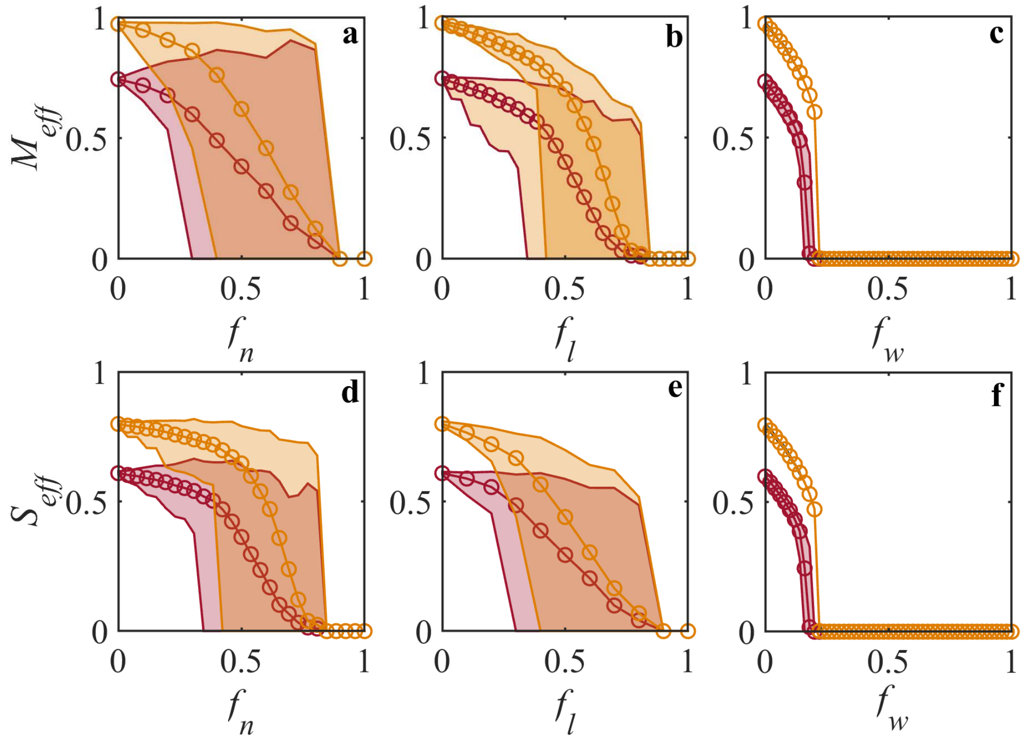

Figure S15: Robustness of dimension reduced model against random parameter variation of β. (a) and (d) show the throughput for manufacturer and supplier when subjected to manufacturer removal and supplier removal respectively. (b) and (e) show the throughput for manufacturer and supplier when subjected to supplier removal and manufacturer removal respectively. (c) and (f) represent the effect of global perturbation on throughput of manufacturer and supplier respectively in form of weight reduction. Maroon color represents multidimensional model (Equations 1 and 2 from the main text), Orange color represents dimension reduced model (Equations 4 and 5 from the main text). The line with circle is the average of 100 realizations and the shaded area shows the lower bound and the upper bound values. $K = 1$, $\alpha = 1.2$, $\gamma_0 = 1$, $\delta = 0.5$, $h = 0.5$. The values of parameter β were subjected to random variations using an uniform distribution $U[0.001, 0.002]$. The synthetic network includes 10 manufacturers and 26 suppliers.

Fig. S16-S28 show the effective throughput M_{eff} of manufacturers and S_{eff} of suppliers in a mutualistic network following a perturbation vs. the perturbation size. We investigate three forms of perturbation for 21 networks Net 1 – Net 21 (Table S1): removal of an f_n fraction of nodes (e.g. manufacturer), perturbing the link through the removal of an f_l fraction of symbiotic interactors (suppliers), reducing all weights by an average of f_w.

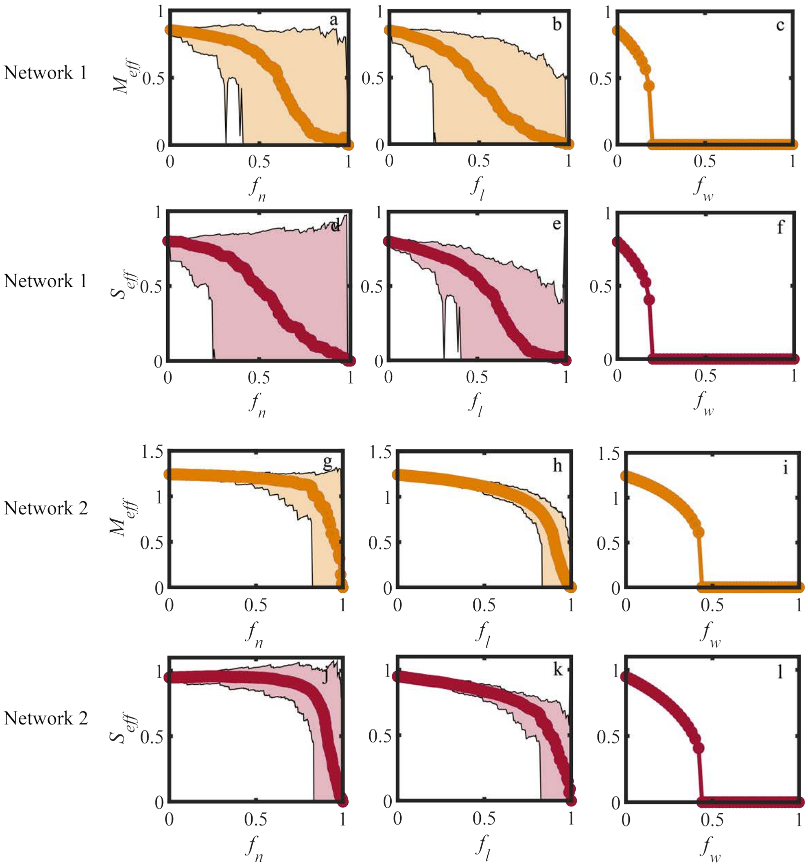

Figure S16: Resilience profile of network 1 and network 2 (see Supplementary Material Table S2). The resiliency profiles were computed using the dimension reduced model (Equations 4 and 5 from the main text). (a) and (d) show the throughput for manufacturer and supplier when subjected to manufacturer removal and supplier removal respectively for network 1. (b) and (e) show the throughput for manufacturer and supplier when subjected to supplier removal and manufacturer removal respectively. (c) and (f) represent the effect of global perturbation on throughput of manufacturer and supplier respectively in the form of weight reduction. The line with circle is the average of 100 realizations and the shaded area shows the lower bound and the upper bound values. $K = 1, \beta = 0.0001, \gamma_0 = 1, \delta = 0.5, h = 0.5$. For network 2, subplots g-l have the same explanation as above for network 1.

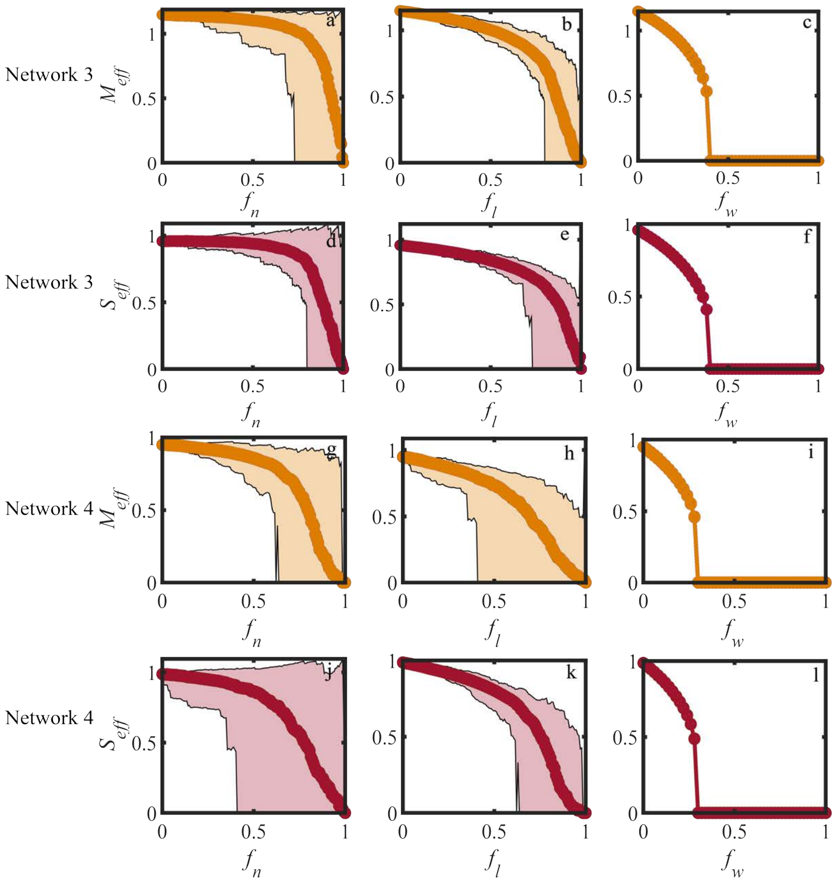

Figure S17: Resilience profile of network 3 and network 4 (see Supplementary Material Table S2). The resiliency profiles were computed using the dimension reduced model (Equations 4 and 5 from the main text). (a) and (d) show the throughput for manufacturer and supplier when subjected to manufacturer removal and supplier removal respectively. (b) and (e) show the throughput for manufacturer and supplier when subjected to supplier removal and manufacturer removal respectively. (c) and (f) represent the effect of global perturbation on throughput of manufacturer and supplier respectively in form of weight reduction. The line with circle is the average of 100 realizations and the shaded area shows the lower bound and the upper bound values. $K = 1$, $\beta = 0.0001$, $\gamma_0 = 1$, $\delta = 0.5$, $h = 0.5$. For network 4, subplots g-l have the same explanation as above for network 3.

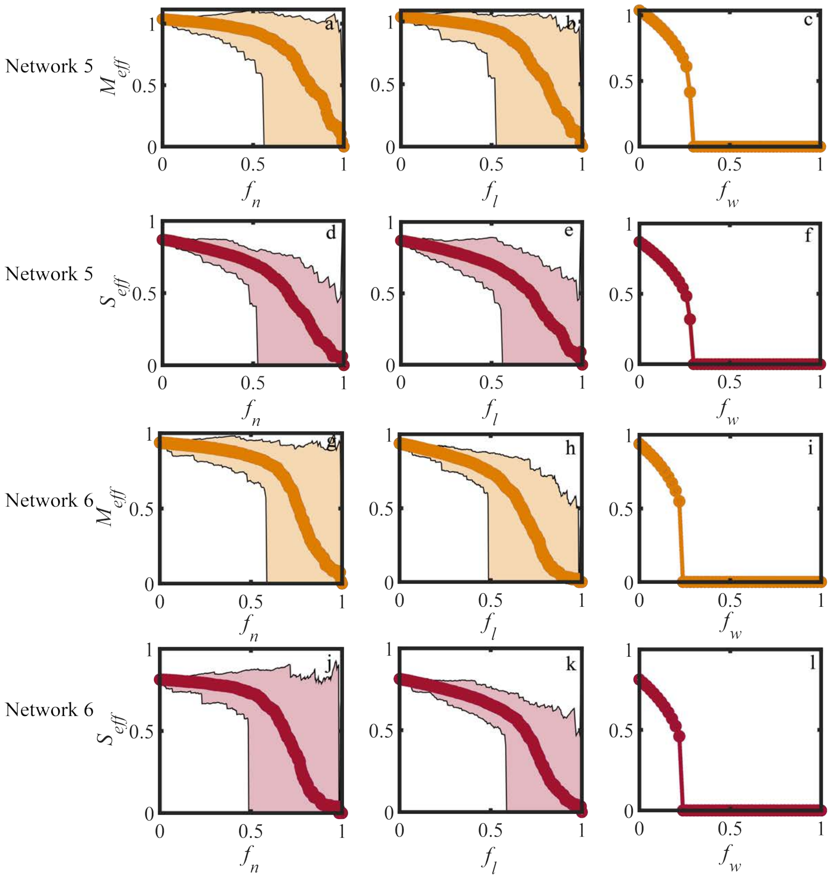

Figure S18: Resilience profile of network 5 and network 6 (see Supplementary Material Table S2). The resiliency profiles were computed using the dimension reduced model (Equations 4 and 5 from the main text). (a) and (d) show the throughput for manufacturer and supplier when subjected to manufacturer removal and supplier removal respectively. (b) and (e) show the throughput for manufacturer and supplier when subjected to supplier removal and manufacturer removal respectively. (c) and (f) represent the effect of global perturbation on throughput of manufacturer and supplier respectively in form of weight reduction. The line with circle is the average of 100 realizations and the shaded area shows the lower bound and the upper bound values. $K = 1$, $\beta = 0.0001$, $\gamma_0 = 1$, $\delta = 0.5$, $h = 0.5$. For network 6, subplots g-l have the same explanation as above for network 5.

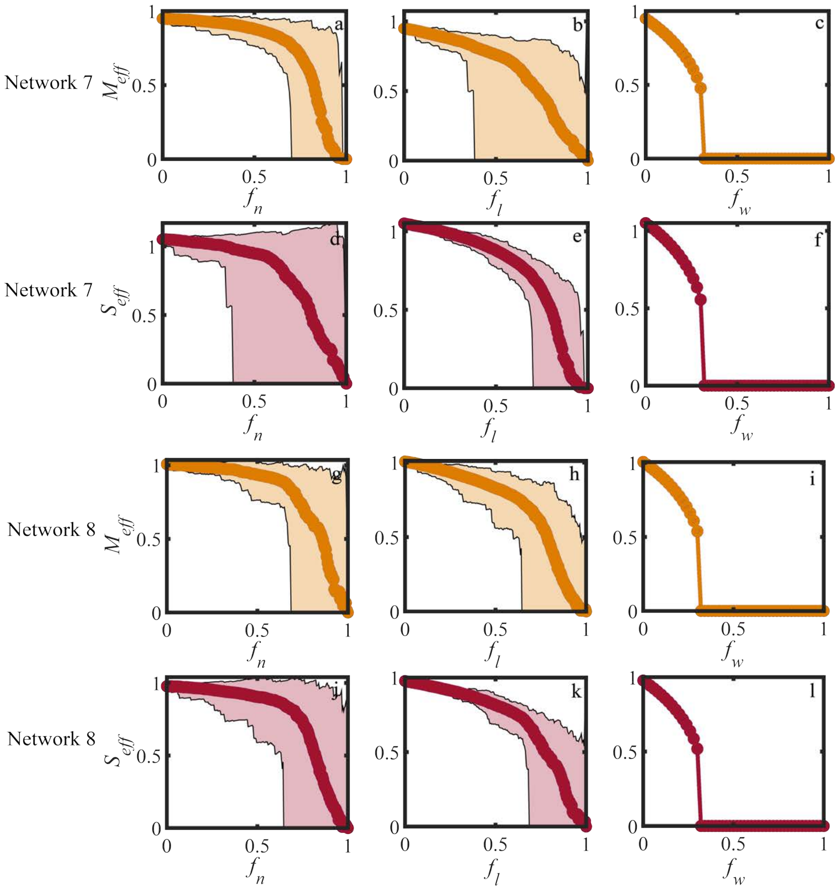

Figure S19: Resilience profile of network 7 and network 8 (see Supplementary Material Table S2). The resiliency profiles were computed using the dimension reduced model (Equations 4 and 5 from the main text). (a) and (d) show the throughput for manufacturer and supplier when subjected to manufacturer removal and supplier removal respectively. (b) and (e) show the throughput for manufacturer and supplier when subjected to supplier removal and manufacturer removal respectively. (c) and (f) represent the effect of global perturbation on throughput of manufacturer and supplier respectively in form of weight reduction. The line with circle is the average of 100 realizations and the shaded area shows the lower bound and the upper bound values. $K = 1$, $\beta = 0.0001$, $\gamma_0 = 1$, $\delta = 0.5$, $h = 0.5$. For network 8, subplots g-l have the same explanation as above for network 7.

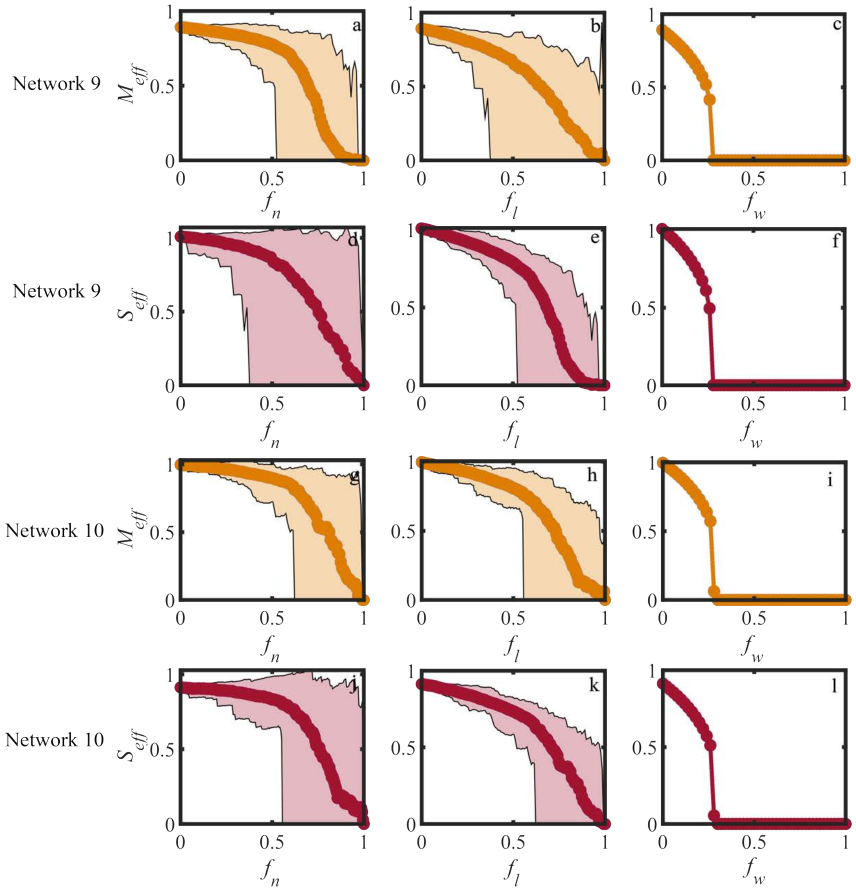

Figure S20: Resilience profile of network 9 and network 10 (see Supplementary Material Table S2). The resiliency profiles were computed using the dimension reduced model (Equations 4 and 5 from the main text). (a) and (d) show the throughput for manufacturer and supplier when subjected to manufacturer removal and supplier removal respectively. (b) and (e) show the throughput for manufacturer and supplier when subjected to supplier removal and manufacturer removal respectively. (c) and (f) represent the effect of global perturbation on throughput of manufacturer and supplier respectively in form of weight reduction. The line with circle is the average of 100 realizations and the shaded area shows the lower bound and the upper bound values. $K = 1$, $\beta = 0.0001$, $\gamma_0 = 1$, $\delta = 0.5$, $h = 0.5$. For network 10, subplots g-l have the same explanation as above for network 9.

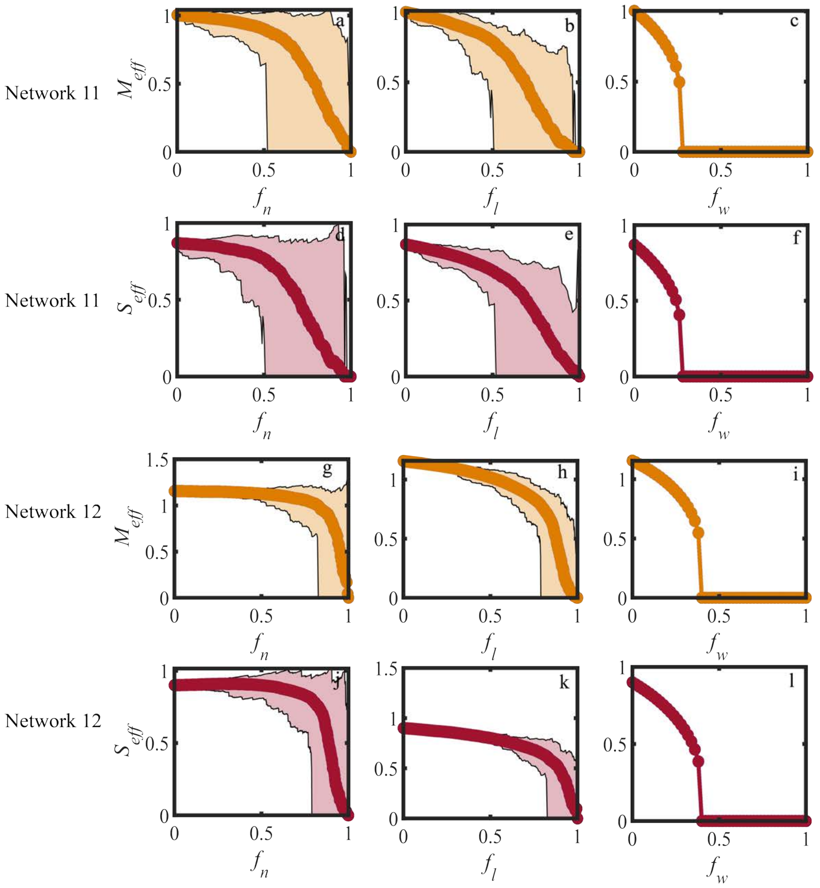

Figure S21: Resilience profile of network 11 and network 12 (see Supplementary Material Table S2). The resiliency profiles were computed using the dimension reduced model (Equations 4 and 5 from the main text). (a) and (d) show the throughput for manufacturer and supplier when subjected to manufacturer removal and supplier removal respectively. (b) and (e) show the throughput for manufacturer and supplier when subjected to supplier removal and manufacturer removal respectively. (c) and (f) represent the effect of global perturbation on throughput of manufacturer and supplier respectively in form of weight reduction. The line with circle is the average of 100 realizations and the shaded area shows the lower bound and the upper bound values. $K = 1$, $\beta = 0.0001$, $\gamma_0 = 1$, $\delta = 0.5$, $h = 0.5$. For network 12, subplots g-l have the same explanation as above for network 11.

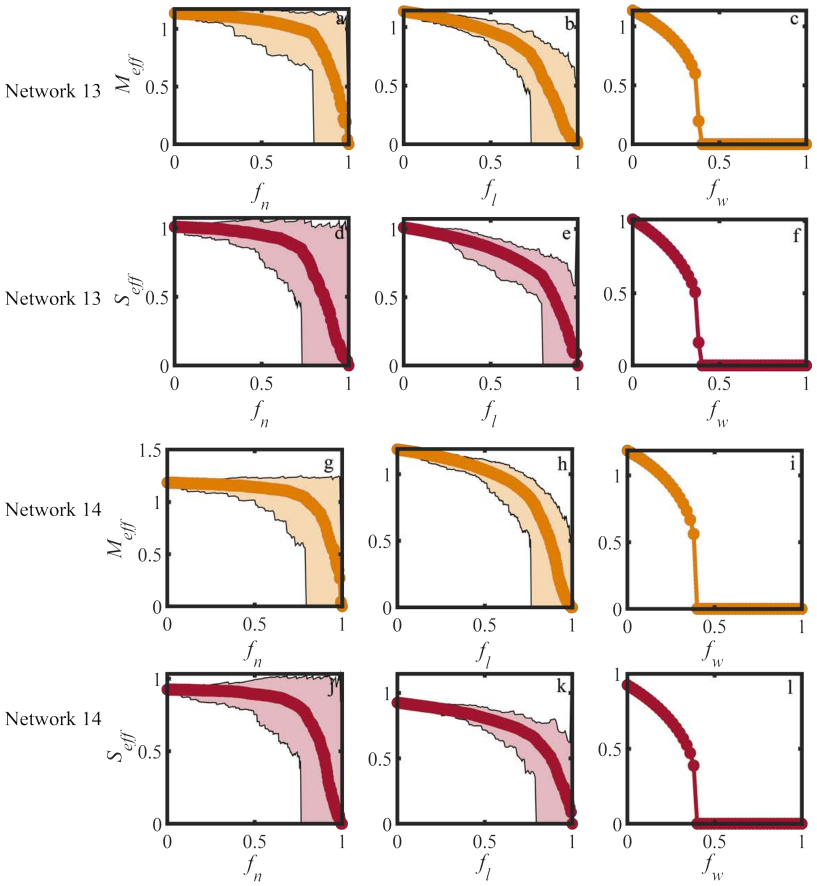

Figure S22: Resilience profile of network 13 and network 14 (see Supplementary Material Table S2). The resiliency profiles were computed using the dimension reduced model (Equations 4 and 5 from the main text). (a) and (d) show the throughput for manufacturer and supplier when subjected to manufacturer removal and supplier removal respectively. (b) and (e) show the throughput for manufacturer and supplier when subjected to supplier removal and manufacturer removal respectively. (c) and (f) represent the effect of global perturbation on throughput of manufacturer and supplier respectively in form of weight reduction. The line with circle is the average of 100 realizations and the shaded area shows the lower bound and the upper bound values. $K = 1$, $\beta = 0.0001$, $\gamma_0 = 1$, $\delta = 0.5$, $h = 0.5$. For network 14, subplots g-l have the same explanation as above for network 13.

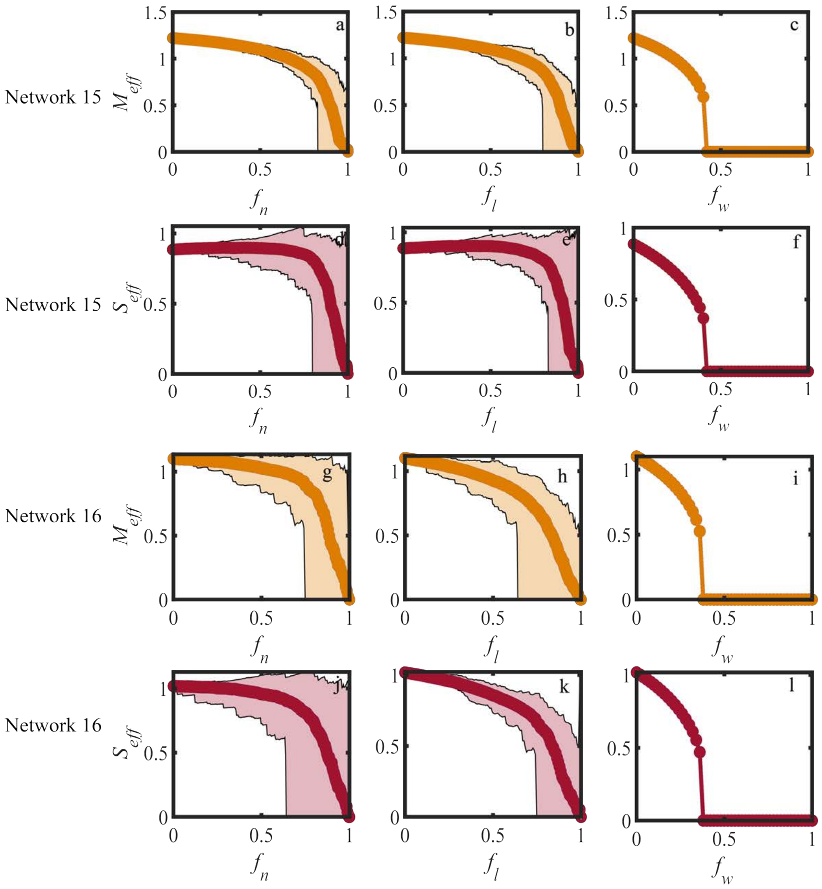

Figure S23: Resilience profile of network 15 and network 16 (see Supplementary Material Table S2). The resiliency profiles were computed using the dimension reduced model (Equations 4 and 5 from the main text). (a) and (d) show the throughput for manufacturer and supplier when subjected to manufacturer removal and supplier removal respectively. (b) and (e) show the throughput for manufacturer and supplier when subjected to supplier removal and manufacturer removal respectively. (c) and (f) represent the effect of global perturbation on throughput of manufacturer and supplier respectively in form of weight reduction. The line with circle is the average of 100 realizations and the shaded area shows the lower bound and the upper bound values. $K = 1$, $\beta = 0.0001$, $\gamma_0 = 1$, $\delta = 0.5$, $h = 0.5$. For network 16, subplots g-l have the same explanation as above for network 15.

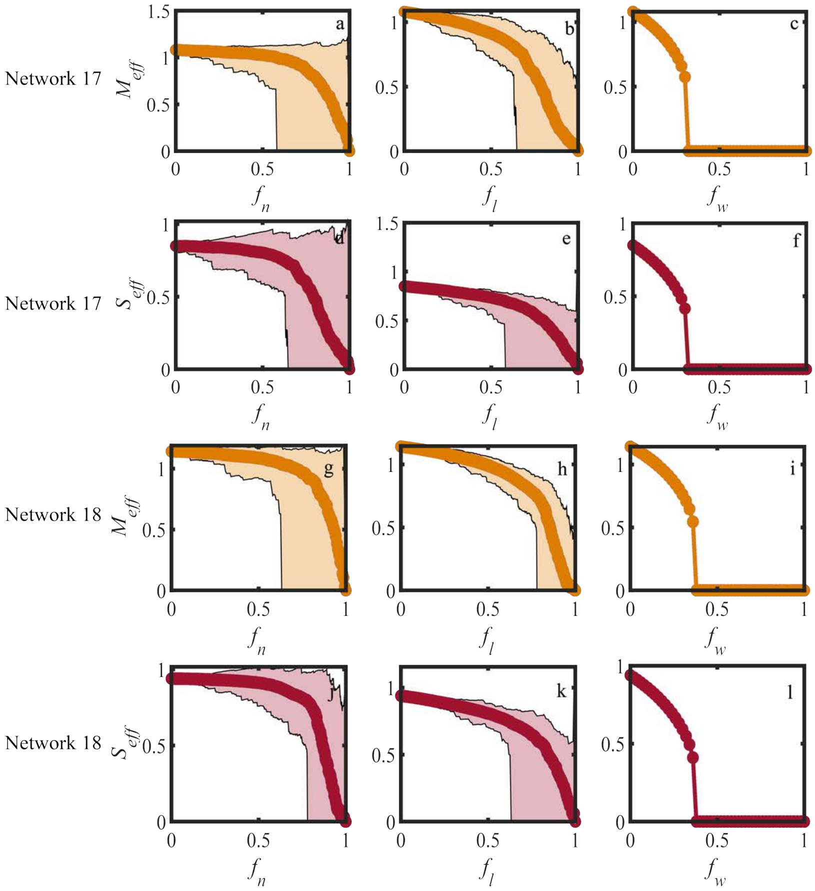

Figure S24: Resilience profile of network 17 and network 18 (see Supplementary Material Table S2). The resiliency profiles were computed using the dimension reduced model (Equations 4 and 5 from the main text). (a) and (d) show the throughput for manufacturer and supplier when subjected to manufacturer removal and supplier removal respectively. (b) and (e) show the throughput for manufacturer and supplier when subjected to supplier removal and manufacturer removal respectively. (c) and (f) represent the effect of global perturbation on throughput of manufacturer and supplier respectively in form of weight reduction. The line with circle is the average of 100 realizations and the shaded area shows the lower bound and the upper bound values. $K = 1$, $\beta = 0.0001$, $\gamma_0 = 1$, $\delta = 0.5$, $h = 0.5$. For network 18, subplots g-l have the same explanation as above for network 17.

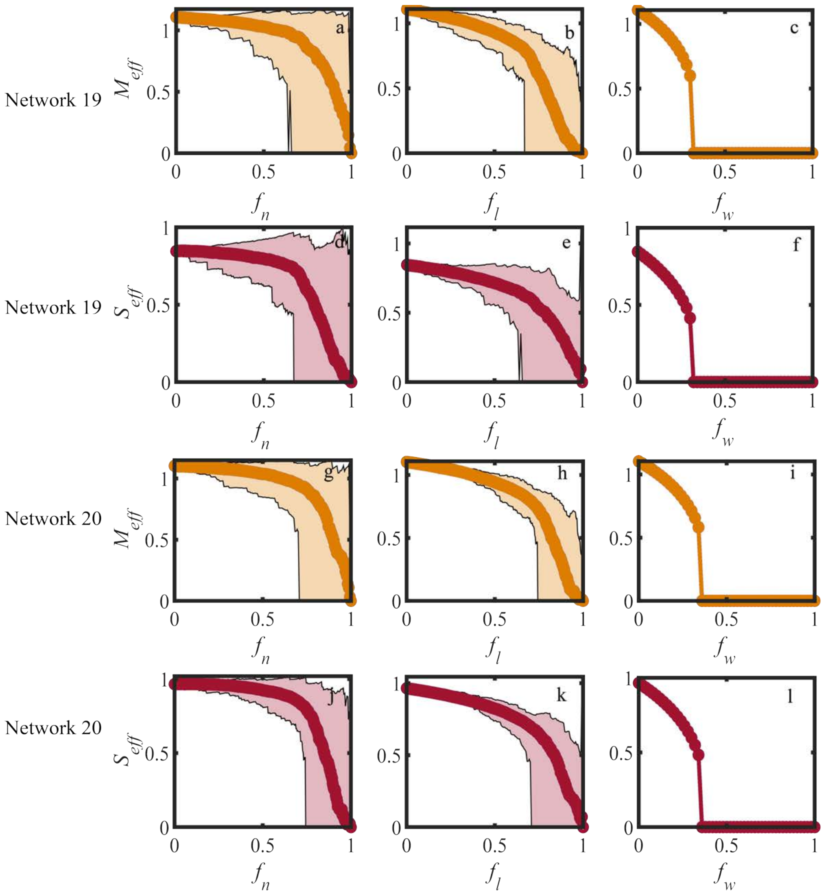

Figure S25: Resilience profile of network 19 and network 20 (see Supplementary Material Table S2). The resiliency profiles were computed using the dimension reduced model (Equations 4 and 5 from the main text). (a) and (d) show the throughput for manufacturer and supplier when subjected to manufacturer removal and supplier removal respectively. (b) and (e) show the throughput for manufacturer and supplier when subjected to supplier removal and manufacturer removal respectively. (c) and (f) represent the effect of global perturbation on throughput of manufacturer and supplier respectively in form of weight reduction. The line with circle is the average of 100 realizations and the shaded area shows the lower bound and the upper bound values. $K = 1$, $\beta = 0.0001$, $\gamma_0 = 1$, $\delta = 0.5$, $h = 0.5$. For network 20, subplots g-l have the same explanation as above for network 19.

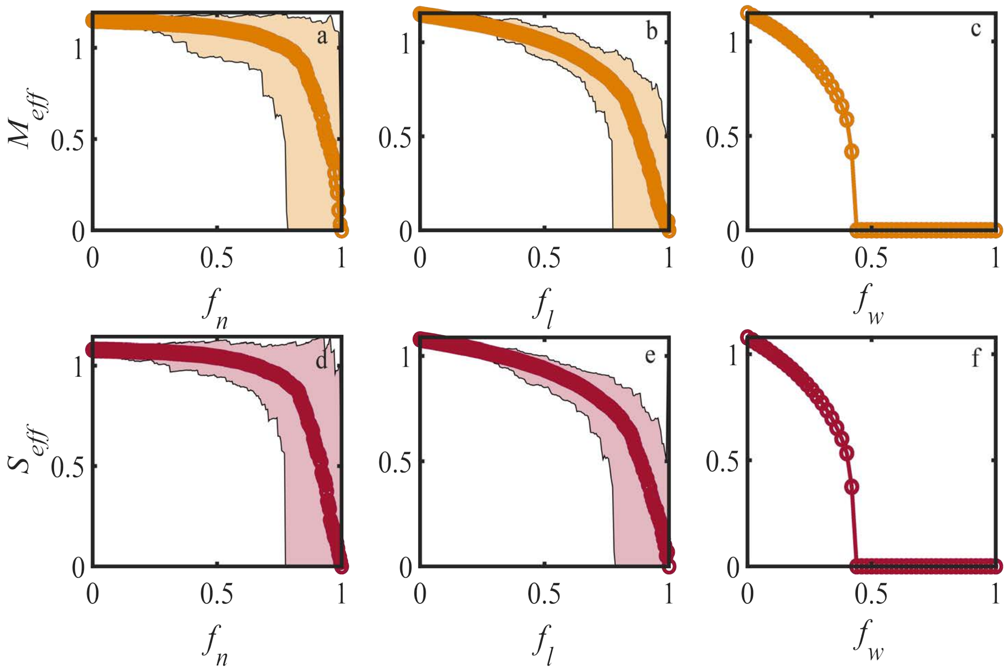

Figure S26: Resilience profile of network 21 (see Supplementary Material Table S2). The resiliency profiles were computed using the dimension reduced model (Equations 4 and 5 from the main text). (a) and (d) show the throughput for manufacturer and supplier when subjected to manufacturer removal and supplier removal respectively. (b) and (e) show the throughput for manufacturer and supplier when subjected to supplier removal and manufacturer removal respectively. (c) and (f) represent the effect of global perturbation on throughput of manufacturer and supplier respectively in form of weight reduction. The line with circle is the average of 100 realizations and the shaded area shows the lower bound and the upper bound values. $K = 1$, $\beta = 0.0001$, $\gamma_0 = 1$, $\delta = 0.5$, $h = 0.5$.

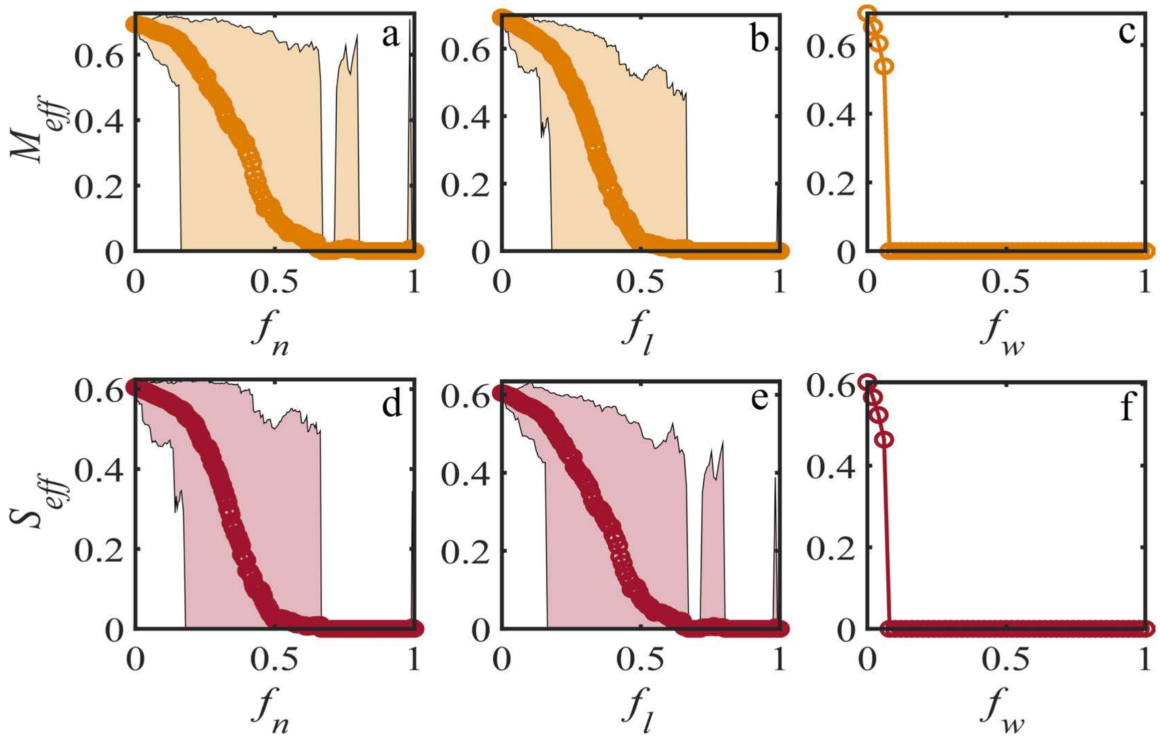

Figure S27: Resilience profile of network 12 for the year 2017 (see Supplementary Material Table S3). The resiliency profiles were computed using the dimension reduced model (Equations 4 and 5 from the main text). (a) and (d) show the throughput for manufacturer and supplier when subjected to manufacturer removal and supplier removal respectively. (b) and (e) show the throughput for manufacturer and supplier when subjected to supplier removal and manufacturer removal respectively. (c) and (f) represent the effect of global perturbation on throughput of manufacturer and supplier respectively in form of weight reduction. The line with circle is the average of 100 realizations and the shaded area shows the lower bound and the upper bound values. $K = 1$, $\beta = 0.0001$, $\gamma_0 = 1$, $\delta = 0.5$, $h = 0.5$.

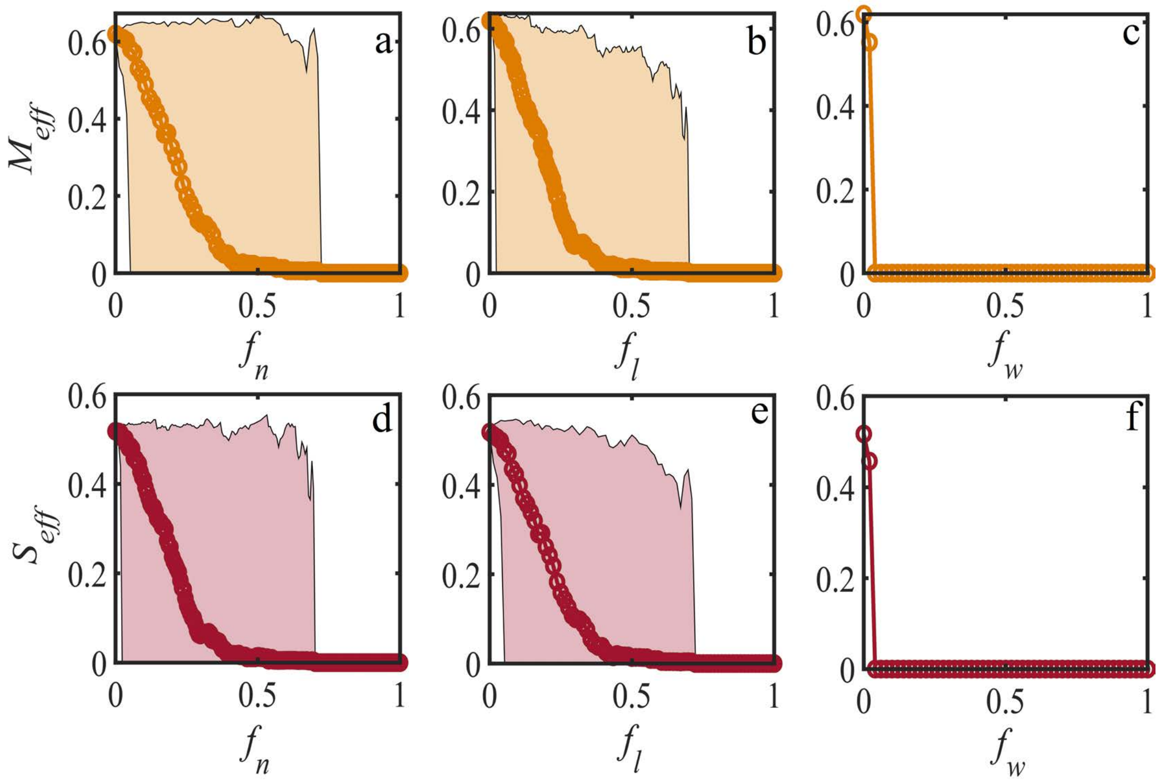

Figure S28: Resilience profile of network 15 for the year 2017 (see Supplementary Material Table S3). The resiliency profiles were computed using the dimension reduced model (Equations 4 and 5 from the main text). (a) and (d) show the throughput for manufacturer and supplier when subjected to manufacturer removal and supplier removal respectively. (b) and (e) show the throughput for manufacturer and supplier when subjected to supplier removal and manufacturer removal respectively. (c) and (f) represent the effect of global perturbation on throughput of manufacturer and supplier respectively in form of weight reduction. The line with circle is the average of 100 realizations and the shaded area shows the lower bound and the upper bound values. $K = 1$, $\beta = 0.0001$, $\gamma_0 = 1$, $\delta = 0.5$, $h = 0.5$.

Table S1: AA Classification of 21 automotive supplier-manufacturer networks

AA Classification	Included Components
Axle/Shaft	Axle
	Drive Shaft
	Propshaft
Body Components	Back Door Lock/Trunk Lock
	Exterior Mirror
	Hinge
	Inside Door Handle
	Pedal
	Roof rail
	Slide Door/Sliding Door Closure
	Tailgate/Trunk Closure
	Window Washer
Drivetrain	All Wheel Drive
	Automatic Transmission
	Clutch Disc
	Cluth Slave Cylinder
	Differential
	Electric All Wheel Drive
	Shift Lever
	Torque Converter
	Transmission Seal
	Automated Manual Transmission
	Clutch
	Clutch Master Cylinder
	CVT
	Dual Clutch Transmission
	Manual Transmission
	Synchronizer
	Transfer
	Transmission Shaft
Engine (Starter/Battery System)	Alternator/Generator
	Battery
	Starter Motor
Engine (Cooling System)	Cooling Fan/Coolin Fan Motor
	Radiator
	Thermostat
	Continued on next page

Table S1 – continued from previous page

AA Classification	Included Components
	Engine Cooling Module
	Radiator Hose
	Water Pump
Engine (Exhaust System)	Diesel Particulate Filter
	Exhaust System
	Exhaust Manifold gasket
	Muffler
	EGR System
	Exhaust Manifold gasket
	Catalytic Converter
Engine (Force Induction System)	Inter Cooler/Charge Air Cooler
	Turbo/Supercharger
Engine (Fuel System)	Carbon Canister
	Fuel Filter
	Fuel Hose
	Fuel Pump
	Engine Management System
	Fuel Filter (Diesel)
	Fuel Line
	Duel Supply System/Module
Engine (Ignition System)	Glow Plug
	Spark Plug
	Ignition Coil
Engine (Intake/Injection System)	Intake Manifold
	Air Cleaner/Air Filter
	Diesel Injection
	Air Intake System
	Throttle Body
	Fuel Injection
Engine (Lubrication System)	Oil Cooler
	Oil Pan
	Oil Strainer/Separator
	Oil Filter
	Oil Pump
Engine (Main Engine Parts)	Connecting Rod
	Cylinder Block
	Cylinder head Cover

Continued on next page

Table S1 – continued from previous page

AA Classification	Included Components
	Cylinder Liner
	Engine Assembly
	Engine Mount
	Piston
	Piston Ring
	V-Belt
	Crankshaft
	Cylinder Head Cover
	Cylinder Head Gasket
	Drive Plate
	Engine Bearing
	Flywheel
	Piston Pin
	Torsional Damper
Engine (Valve Train System)	Camshaft
	Rocker Arm
	Timing System
	Valve Spring
	Engine Valve
	Timing Belt/Timing Chain
	Valve Guide/Valve Seat
Exterior	Bumper
	Glass Run Channel
	Molding (Roof, etc.)
	Spoiler
	Wheel Cover/Cap
	Emblem
	Molding (Body Side)
	Radiator Grille
	Weatherstrip
	Window Glass
Interior	Ashtray
	Console
	Cup Holder
	Display
	Door trim
	Floor Mat

Continued on next page

Table S1 – continued from previous page

AA Classification	Included Components
	Headliner
	Interior Lighting
	Meter
	Trunk/Tailgate trim
	Cockpit Module
	Corss Car Beam
	Dash Panel
	Door Panel
	Floor Carpet
	Glove Box
	Instrumrument Panel
	Interior Mirror
	Sun Visor
lamp/Wiper	Exterior Lighting
	Head Lamp
	Head Lamp Cleaner
	Rear Lamp
	Fog Lamp
	Head Lamp (AFS)
	High-Mounted Stop Lamp
	Wiper System
Seats	Headrest
	Seat Adjuster/Recliner
	Seat Lumbar Support
	Seat
	Seat Frame
	Seat Trim
Steering	Key Cylinder/Steering Lock
	Power Steering Hose
	Power Steering Pump
	Steering Column/Shaft
	Steering Knuckle
	Steering Wheel
	Power Steering Assist Unit
	Power Steering Motor
	Rack End
	Steering Gear

Continued on next page

Table S1 – continued from previous page

AA Classification	Included Components
	Steering System
	Tie Rod End
Structure/Module/Body/Others	Bumper Beam
	Crossmember
	Door Module
	Fuel Tank
	Side Impact Beam
	Chassis Frame
	Door Frame
	Front End Module
	Heat Sheild
Suspension/Subframe	Shock Absorber
	Subframe/Suspension member
	Suspension Control Arm
	Suspension Spring
	Stabilizer
	Suspension Ball Joint
	Suspension Module/System
Wheel/Tyre	Tyre
	Wheel Bearing
	Wheel

Table S2: The 21 automotive supplier-manufacturer networks are from Marklines database

Network No.	Secondary Classification	# Manufacturer	# Supplier	Nestedness	Density	Year
1	Axle/Shaft	115	189	0.9863	0.0226	2006-2020
2	Body Components	166	603	0.9930	0.0192	1999-2019
3	Drivetrain	186	488	0.9949	0.0177	2006-2019
4	Engine (Starter/Battery System)	135	148	0.9901	0.0287	2001-2018
5	Engine (Cooling System	116	264	0.9856	0.0251	2002-2018
6	Engine (Exhaust System)	189	413	0.9897	0.0149	2006-2019
7	Engine (Forced Induction System)	158	138	0.9843	0.0309	2006-2020
8	Engine (Fuel System)	188	253	0.9903	0.0210	2006-2019
9	Engine (Ignition system)	101	69	0.9757	0.0496	2002-2018
10	Engine (Intake/Injection System)	127	222	0.9878	0.0237	2006-2019
11	Engine (Lubrication system)	100	221	0.9856	0.0272	2006-2018
12	Engine (Main Engine Parts)	219	749	0.9926	0.0154	2006-2019
13	Engine (Valve Train System)	130	253	0.9888	0.0297	2006-2019
14	Exterior	146	501	0.9911	0.0203	2006-2019
15	Interior	172	780	0.9941	0.0161	2006-2019
16	Lamp/Wiper	139	259	0.9869	0.0269	2001-2019
17	Seats	165	517	0.9933	0.0148	2004-2020
18	Steering	155	420	0.9923	0.0208	2006-2019
19	Structure/Module/Body	117	447	0.9888	0.0202	2006-2019
20	Suspension/Subframe	164	346	0.9917	0.0213	2006-2020
21	Wheel/Tyre	181	266	0.9876	0.0292	2006-2020

Table S3: Supplier-manufacturer networks from Marklines database for the year 2017

Network No.	Secondary Classification	# Manufacturer	# Supplier	Nestedness	Density
12	Engine (Main Engine Parts)	122	268	0.9756	0.0192
15	Interior	77	172	0.9788	0.0255

References

[1] F. Morone, G. Del Ferraro, H. A. Makse, The k-core as a predictor of structural collapse in mutualistic ecosystems, Nature Physics 15 (2019) 95.

[2] J. Gao, B. Barzel, A.-L. Barabási, Universal resilience patterns in complex networks, Nature 530 (2016) 307.

[3] J. N. Holland, D. L. DeAngelis, J. L. Bronstein, Population dynamics and mutualism: functional responses of benefits and costs, The American Naturalist 159 (2002) 231–244.

[4] J. Jiang, Z.-G. Huang, T. P. Seager, W. Lin, C. Grebogi, A. Hastings, Y.-C. Lai, Predicting tipping points in mutualistic networks through dimension reduction, Proceedings of the National Academy of Sciences 115 (2018) E639–E647.

[5] S. Saavedra, F. Reed-Tsochas, B. Uzzi, A simple model of bipartite cooperation for ecological and organizational networks, Nature 457 (2009) 463.

[6] A. Brintrup, T. Kito, A. Alzayed, M. Meyer, Nested patterns in large-scale automotive supply networks, Capturing Value Int. Manuf. Supply Networks, Institute for Manufacturing (2012).

[7] A. Brintrup, J. Barros, A. Tiwari, The nested structure of emergent supply networks, IEEE Systems Journal (2015) 1–10.

[8] M. McCarthy, The allee effect, finding mates and theoretical models, Ecological Modelling 103 (1997) 99–102.

[9] C. Marchetti, The automobile in a system context: The past 80 years and the next 20 years, Technological Forecasting and Social Change 23 (1983).

[10] M. Höök, J. Li, N. Oba, S. Snowden, Descriptive and predictive growth curves in energy system analysis, Natural Resources Research 20 (2011) 103–116.

[11] T. Modis, A. Debecker, Chaoslike states can be expected before and after logistic growth, Technological forecasting and social change 41 (1992) 111–120.
